# Adipokines in Obese Asthma: A Complex Relationship Influenced More by Sex, Weight, and Oral Steroid Treatment Than Disease Severity

**DOI:** 10.1111/all.70318

**Published:** 2026-04-03

**Authors:** Lars I. Andersson, Maciej Kupczyk, Barbro Dahlén, Kenji Izuhara, Mina Gaga, Nikos M. Siafakas, Alberto Papi, Bianca Beghe, Guy Joos, Klaus F. Rabe, Elisabeth H. Bel, Sebastian L. Johnston, Pascal Chanez, Mark Gjomarkaj, Peter H. Howarth, Ewa Niżankowska‐Mogilnicka, Roelinde Middelveld, Sven‐Erik Dahlén, Apostolos Bossios, Anna James, the BIOAIR (Longitudinal Assessment of Clinical Course and BIOmarkers in Severe Chronic AIRway Disease) and ChAMP consortia

**Affiliations:** ^1^ Clinical Lung‐ and Allergy Research Unit, Department of Medicine Huddinge Karolinska Institutet Stockholm Sweden; ^2^ Department of Respiratory Medicine and Allergy Karolinska University Hospital Huddinge Stockholm Sweden; ^3^ Department of Internal Medicine, Asthma and Allergy Medical University of Lodz, University of Lodz Lodz Poland; ^4^ Division of Medical Biochemistry, Department of Biomolecular Sciences Saga Medical School Saga Japan; ^5^ Respiratory Medicine Dept and Asthma Centre, Athens Chest Hospital “Sotiria”, University of Athens Athens Greece; ^6^ Department of Thoracic Medicine Medical School, University of Crete Heraklion Greece; ^7^ Division of Internal and Cardiorespiratory Medicine University of Ferrara Ferrara Italy; ^8^ Department of Medical and Surgical Sciences University of Modena and Reggio Emilia Modena Italy; ^9^ Department of Internal Medicine and Pediatrics Ghent University Ghent Belgium; ^10^ Department of Respiratory Medicine Ghent University Hospital Ghent Belgium; ^11^ Department of Internal Medicine Christian Albrecht's University Kiel Kiel Germany; ^12^ Department of Respiratory Medicine, Amsterdam UMC University of Amsterdam Amsterdam the Netherlands; ^13^ National Heart and Lung Institute, Imperial College London London UK; ^14^ Assistance Publique des Hôpitaux de Marseille, Clinique des Bronches, Allergies et Sommeil, Aix Marseille Université Marseille France; ^15^ Institute of Translational Pharmacology, Italian National Research Council Palermo Italy; ^16^ Global Medical Affairs, Respiratory Biologics, Specialty Medicine, GSK London UK; ^17^ NIHR Southampton Biomedical Research Centre, University Hospital Southampton NHS Foundation Trust, and Clinical and Experimental Sciences, Faculty of Medicine University of Southampton Southampton UK; ^18^ Department of Internal Medicine Jagiellonian University Medical College Krakow Poland; ^19^ Department of Physiology and Pharmacology Karolinska Institutet Stockholm Sweden; ^20^ Unit of Integrative Metabolomics, Institute of Environmental Medicine, Karolinska Institutet Stockholm Sweden; ^21^ Karolinska Severe Asthma Centre Karolinska University Hospital Huddinge Stockholm Sweden; ^22^ Division for Lung and Airway Research Institute of Environmental Medicine, Karolinska Institutet Stockholm Sweden; ^23^ Lung Laboratory, Center for Molecular Medicine Karolinska University Hospital Stockholm Sweden; ^24^ Institute of Women's and Children's Health, Karolinska Institutet Stockholm Sweden

**Keywords:** adipokines, asthma, biomarkers, obesity

## Abstract

**Background:**

Obesity‐related asthma (OBA) is a distinct asthma phenotype, with increased severity. Adipokine release from excessive adipose tissue is suggested to be a key feature of OBA pathophysiology. However, it is unclear how the clinical characteristics of severe asthma associate with adipokine mediators. We examined systemic adipokine levels and evaluated relationships with disease severity, weight, sex, and steroid treatment in asthma.

**Methods:**

A multiplex immunoassay for nine adipokines with proposed involvement in obesity‐related inflammation (adiponectin, adipsin, BAFF, chemerin, FGF‐21, leptin, lipocalin‐2/NGAL, osteonectin and resistin) was designed. Plasma adipokines were measured in 127 patients with mild‐to‐moderate asthma (MMA) or severe asthma (SA) from the European BIOAIR cohort at baseline and after a controlled 2‐week oral corticosteroid (OCS) intervention.

**Results:**

Leptin and chemerin were significantly increased in patients with SA vs. MMA. Leptin, adiponectin, adipsin, and NGAL were affected by sex, whereas leptin and adipsin were strongly affected by weight. OCS increased leptin and adiponectin, decreased adipsin and BAFF, and did not affect osteonectin, resistin, or chemerin. No adipokines showed positive associations with exhaled NO, blood or sputum eosinophils, although certain correlations with serum CRP, blood, and sputum neutrophils were observed.

**Conclusions:**

Overall, we observe variable relationships between the nine adipokines, obesity and asthma severity. There were no relationships between adipokine levels and type‐2 airway inflammation, yet associations with systemic neutrophilic inflammation were seen. Although one adipokine, chemerin, was independently associated with asthma severity, deciphering the role of adipokines in OBA is complex due to the influence of sex, BMI, and OCS.

## Graphical abstract

Adipokine secretion from adipose tissue may be involved in obesity‐related asthma, but it is unknown how adipokine levels relate to asthma characteristics. Plasma adipokines were measured in well‐characterized lean, overweight or obese patients with mild‐to‐moderate or severe asthma. Adipokine levels were most strongly influenced by sex, oral corticosteroids, and weight status, and deciphering their role in obesity‐related asthma is complex. BMI, body mass index; BAFF, B‐cell activating factor; FGF‐21, fibroblast growth factor 21.

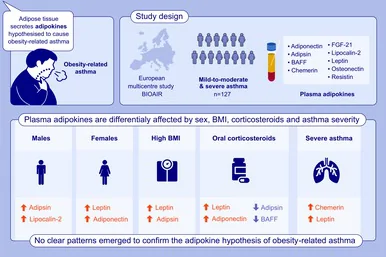

**Abbreviations**
ACQ: Asthma Control Questionnaire
BAFF: B cell activating factor
BIOAIR: longitudinal assessment of clinical course and biomarkers in severe chronic airway diseases
BMI: body mass index
CRP: C‐reactive protein
FeNO: fractional exhaled nitric oxide
FEV_1_
: forced expiratory volume in 1 s
FGF‐21: fibroblast growth factor 21
FVC: forced vital capacity
hsCRP: high sensitivity C‐reactive protein
ICS: inhaled corticosteroid(s)
IL: interleukin
IQR: inter quartile range
LABA: long‐acting beta‐agonists
MMA: mild‐to‐moderate asthma
NGAL: neutrophil gelatinase‐associated lipocalin
OBA: obesity‐related asthma
OCS: oral corticosteroid(s)
SABA: short‐acting beta‐agonists
SA: severe asthma
SGRQ: St. George's Respiratory Questionnaire
WBC: white blood cell

## 1 Introduction

Obesity‐related asthma (OBA) represents a distinct asthma phenotype, with increased severity [1]. Obese patients with asthma have more symptoms, more severe and frequent exacerbations, display a reduced response to asthma medications, and accordingly experience a reduced quality of life [2, 3]. Furthermore, several independent clustering efforts have identified an asthma phenotype characterized by over‐representation of female, obese, severe asthmatic patients [4]. It is debated whether obesity contributes to the development of asthma, or whether it is a consequence of reduced physical activity due to asthma. OBA likely stems from both mechanical factors, where excess adipose tissue restricts lung function, and biological mechanisms that enhance airway hyperreactivity, though the relative contribution of each remains to be elucidated.

Circumstantial evidence suggests that mediators released from adipose tissue induce low‐grade systemic inflammation which might contribute to airway inflammation [5], but this hypothesis derives more from experimental studies than observations in patients. In addition to being a storage depot for energy, adipose tissue is an active endocrine and immune organ. It has a diverse cellular composition capable of secreting numerous proteins, collectively referred to as adipokines [6]. Obesity modifies adipose tissue cellular composition and function [7], which can be reflected by altered lung function alongside differences in patterns of secreted adipokines but the relationship between such changes in humans is unclear [8, 9].

Previous work has shown that plasma levels of the pro‐inflammatory leptin increase with both increased BMI and with asthma severity [10]. In contrast, adiponectin has been suggested to be an anti‐inflammatory signal, with changes in the ratio between leptin and adiponectin modulating systemic inflammation, especially in females [11]. However, studies on the role of leptin, adiponectin, and other adipokines in asthma are in their infancy, mostly consisting of small observational studies, often not focused on severe asthma and involving measurements of one or two adipokine mediators.

This study was therefore initiated with the aim to use a well‐characterized and relatively large cohort of asthmatics to measure nine adipokine mediators, proposed to be involved in OBA based on previous hypotheses (Table 1), and establish how disease severity, sex, obesity, and treatment with oral steroids affect different adipokines. The influence of glucocorticosteroids on adipokine levels may in fact be a confounding factor that is often overlooked.

**TABLE 1:** Summary of adipokines measured in this study.

Adipokine	Cell source	Main function	Pro/anti‐inflammatory?	Association with obesity	Asthma involvement	Ref
Adiponectin	Adipocytes	Improves insulin sensitivity, regulates glucose and lipid metabolism	Anti‐inflammatory	Lower	Protective against airway inflammation	[12, 13]
Adipsin	Adipocytes, macrophages	Complement factor D (activates alternative complement), lipid metabolism	Pro‐inflammatory	Higher	Limited evidence, possible involvement in Th17‐mediated inflammation.	[13, 14, 15]
BAFF	Myeloid cells, adipocytes	Promotes B‐cell survival and antibody production	Pro‐inflammatory	Higher	Implicated in allergic airway inflammation	[16, 17, 18]
Chemerin	Adipocytes, hepatocytes, stromal cells	Immune cell chemotaxis, adipogenesis	Both, context dependent	Higher	Limited evidence, involvement via effects on immune cells	[13, 19]
FGF‐21	Hepatocytes, adipocytes, skeletal muscle	Regulates glucose and lipid metabolism, metabolic stress response	Anti‐inflammatory	Higher	Possible biomarker of asthma/mast cell activation	[13, 20, 21]
Leptin	Adipocytes	Regulates appetite, energy balance, immune activation	Pro‐inflammatory	Higher	Promotes airway inflammation and asthma severity	[13, 22, 23]
Lipocalin‐2 (NGAL)	Adipocytes, neutrophils, macrophages	Innate immunity, iron transport, hematopoietic cell apoptosis	Pro‐inflammatory	Higher	Limited information, associated with neutrophilic asthma	[13, 24]
Osteonectin (SPARC)	Adipocytes, fibroblasts, osteoblasts	Cellular adhesion, tissue remodeling, and fibrosis	Pro‐inflammatory, pro‐fibrotic	Higher	Limited evidence, involved in airway remodeling	[13, 25]
Resistin	Peripheral blood mononuclear cells (PBMCs), macrophages	Promotes insulin resistance, stimulates pro‐inflammatory cytokine release	Pro‐inflammatory	Higher	Higher levels associated with greater response to steroids	[13, 26]

The panel of adipokine mediators was selected based on involvement in biological pathways of relevance to obesity and inflammation, production by both adipocytes and immune and structural cell types outside adipose tissue depots, and known involvement in energy metabolism and insulin resistance, namely: adiponectin, adipsin, BAFF (B cell activating factor), chemerin, FGF‐21 (fibroblast growth factor 21), leptin, lipocalin‐2 (also known as NGAL, Neutrophil Gelatinase‐Associated Lipocalin), osteonectin (also known as SPARC, Secreted Protein Acidic and Rich in Cysteine), and resistin. Their levels were measured in patients with asthma taking part in the European multi‐center study BIOAIR (Longitudinal Assessment of Clinical Course and Biomarkers in Severe Chronic Airway Diseases). The BIOAIR cohort includes 127 patients with mild‐to‐moderate (MMA) and severe asthma (SA), where all patients underwent a double‐blind, placebo‐controlled, oral steroid intervention [27, 28, 29, 30, 31]. About a third of the patients were lean and about a quarter were obese. As participants were followed up over a year with repeated sampling it was also possible to assess internal stability of observations.

Thus, based on the strengths of the BIOAIR cohort, we aimed to provide a basis for further studies attempting to disentangle the complex mechanisms underlying OBA by documenting how key factors known to associate with this phenotype affect levels of circulating adipokines.

## 2 Methods

### 2.1 Study Design and Subjects

Plasma samples were obtained from patients taking part in BIOAIR, a European multicentre study [27, 28, 29, 30, 31]. All subjects were 18–80 years of age and had either mild‐to‐moderate asthma (MMA) or severe asthma (SA). Further details and definitions regarding the BIOAIR study and patient groups are provided in the online supplement. Briefly, patients with MMA had stable disease, received daily treatment with max 800 μg/day budesonide or beclomethasone, 500 μg/day fluticasone or equivalent. MMA subjects used SABA as needed but did not require treatment with LABA and had no exacerbations, nor hospitalisations in the past year. SA patients, on the other hand, had been under specialist treatment for at least 1 year and had experienced at least one exacerbation in the past year. SA patients also required continuous treatment with high doses of ICS (at least 1600 μg/day budesonide or beclomethasone, 800 μg/day fluticasone or equivalent). For those taking oral steroids, the inhaled dose of steroids had to be at least 800 μg/day budesonide or beclomethasone, 400 μg/day fluticasone or equivalent. In addition, SA patients required continuous treatment with LABA or oral theophylline. Following an initial screening visit (visit 1, recruitment), patients followed a 4‐week treatment optimization period specific for each of the two groups as outlined in supplemental information. All analyses in the current investigation were performed using data collected at visit 2, after optimization, as this data was considered less prone to variability caused by differences in medication. Following optimization, patients from all groups took part in a 2‐week, double‐blind, placebo‐controlled oral steroid intervention, consisting of a standard course of prednisolone (0.5 mg/kg of body weight/day) added to regular treatment. The BIOAIR study was approved by the ethics committee of each participating clinical institution and participants provided written informed consent.

### 2.2 Clinical and Laboratory Measurements

Lung function measurements, fraction of exhaled nitric oxide (FeNO) measurements, high sensitivity C‐reactive protein (hsCRP), white blood cell (WBC) counts, periostin analysis, skin prick testing, sputum induction and processing were all performed as described in the online supplement.

### 2.3 BMI Grouping

In order to examine the effect of BMI on airway disease, subjects were divided into three categories: lean (BMI = 18.5–24.9 kg/m^2^), overweight (BMI = 25–29.9 kg/m^2^) and obese (BMI ≥ 30 kg/m^2^). In total, two patients in the BIOAIR study were underweight (BMI < 18.5 kg/m^2^) and these were not included in analyses related to BMI.

### 2.4 Adipokine Analysis

A multiplex panel of mediators of relevance to airway inflammation, obesity and metabolism was designed to measure adiponectin, adipsin, BAFF, FGF‐21, leptin, NGAL, osteonectin, resistin and chemerin (Table S1) in plasma samples. Measurements were performed using a BIORAD, Bio‐Plex 200 Luminex system with screening assay reagents from R&D Systems (Bio‐Techne, Abingdon, UK) and analyzed according to the manufacturers' instructions. Results are expressed as relative fluorescence intensity (RFU).

### 2.5 Statistics

Statistics were performed using GraphPad Prism statistical software (GraphPad software v9, LA, Jolla, CA) and Stata 13 software (StataCorp LP, Texas, USA). Most variables in this study were not normally distributed according to the Kolmogorov–Smirnov test and were therefore analyzed using non‐parametric tests (Mann Whitney for un‐paired data, Wilcoxon for paired, and Kruskal–Wallis for multiple group comparisons) unless otherwise stated in figure legends. Univariate relationships between adipokines and clinical characteristics were examined by Spearman rank tests, and multivariate relationships were examined by regression analysis following log transformation of data. A *p* value < 0.05 was considered statistically significant.

## 3 Results

### 3.1 Effect of Obesity on Clinical Characteristics

**FIGURE 1:**
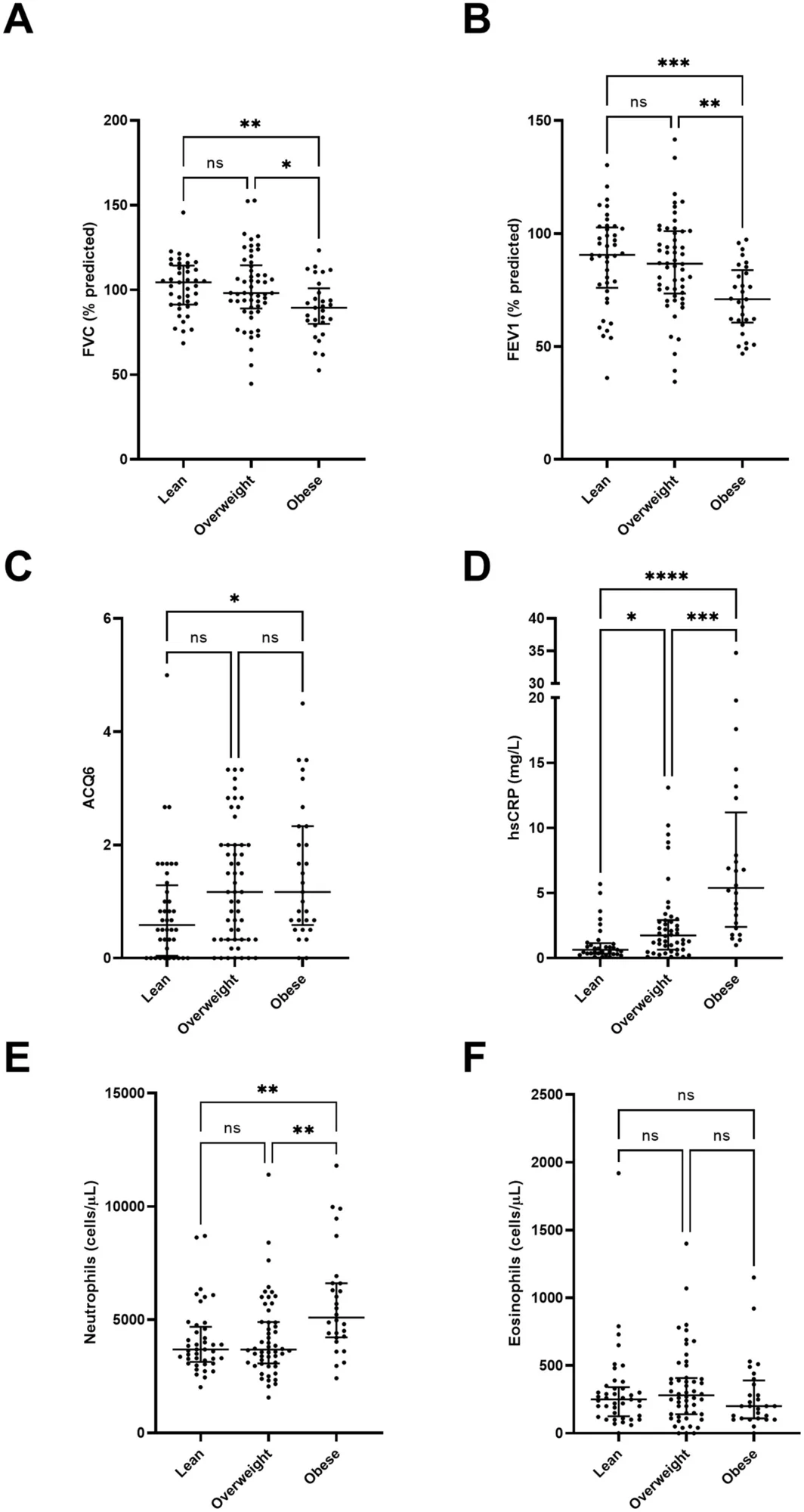
Clinical characteristics of patients with asthma stratified by weight status. Graphs show (A) FVC, (B) FEV_1_, (C) ACQ6, (D) hsCRP, (E) blood neutrophils and (F) blood eosinophils where results are shown as individual data points with median and inter‐quartile range (Kruskal–Wallis test with Dunn's correction for multiple comparisons, *p < 0.05, **p < 0.01, ***p < 0.001, and ****p < 0.0001).

The proportion of overweight and obese individuals was higher in SA than in MMA (Table 2). The obese group showed reduced FVC and FEV_1_ (Figure 1A,B), worse patient‐reported asthma control (ACQ6) (Figure 1C), increased systemic inflammation (hsCRP) (Figure 1D), and higher blood neutrophils (Figure 1E) compared with overweight and lean patients. Increased BMI was not associated with any significant effects on blood eosinophils (Figure 1F), FeNO, sputum neutrophils, sputum eosinophils, or periostin (Table S2). Univariate correlation analyses showed an association between higher BMI with increased hsCRP, blood neutrophils, and ACQ6 scores, and lower measures of FEV_1_ and FVC (Table S3).

**TABLE 2:** Baseline characteristics of patients with mild‐to‐moderate and severe asthma.

	Mild to moderate asthma (MMA)	*N*	Severe asthma (SA)	*N*	Between group comparisons
	Median (IQR)		Median (IQR)		MMA vs. SA
Age (years)	41 (31–50)	55	52 (41–59)	72	0.0004
Sex (M = male), *n* (%)	21 M (38.2%)	55	30 M (41.7%)	72	ns
BMI (kg/m^2^)	24.6 (22.4–27.2)	55	27.8 (25.6–31.5)	72	< 0.0001
Lean, overweight & obese, *n* (%)	30 (54.5%) lean, 19 (34.5%) overweight & 6 (11%) obese	55	14 (19%) lean, 35 (49%) overweight & 23 (32%) obese	72	< 0.0001
Age of onset (years)	20 (5–33)	49	32 (18–44)	61	0.0044
% early (< 12 years of age) or late onset (≥ 12 years of age)	33% early, 67% late	49	15% early, 85% late	61	0.026
Atopy, *n* (%)	37/51 (72.5%)	51	42/70 (60%)	70	ns
FVC (% predicted)	106 (95–116)	53	92 (78–103)	72	< 0.0001
FEV_1_/FVC	0.74 (0.66–0.80)	53	0.69 (0.61–0.78)	72	ns
FEV_1_ (% predicted)	94 (80–102)	53	76 (62–89)	72	< 0.0001
Post‐bronchodilator reversibility (change in FEV_1_)	9.9%	55	8.9%	71	ns
ACQ6 (score)	0.33 (0.0–0.83)	54	1.67 (0.83–2.67)	69	< 0.0001
SGRQ (score)	16.8 (6.8–32.5)	45	44.0 (34.2–56.7)	65	< 0.0001
Blood eosinophils (cells/µL)	250 (135–350)	53	250 (110–465)	69	ns
Blood neutrophils (cells/µL)	3560 (3110–4325)	53	4700 (3590–6230)	69	0.0003
hsCRP (mg/L)	1.1 (0.4–2.6)	47	2.3 (0.9–5.1)	57	0.019
FeNO (ppb)	22 (15–35)	28	30 (12–63)	34	ns
Sputum neutrophils (%)	38 (14–66)	38	45 (24–69)	47	ns
Sputum eosinophils (%)	1 (0.2–4.2)	38	3 (0.8–14.3)	47	0.046
Periostin (ng/mL)	75 (65–91)	50	79 (62–104)	68	ns
Adiponectin (RFU)	5110 (4200–5860)	55	5145 (3970–5930)	72	ns
Adipsin (RFU)	3570 (3010–4160)	55	3640 (3140–4400)	72	ns
BAFF (RFU)	233 (208–268)	55	242 (193–280)	72	ns
Chemerin (RFU)	103 (90.8–124)	42	120 (101–162)	59	0.0053
FGF‐21 (RFU)	135 (117–170)	55	142 (123–164)	72	ns
Leptin (RFU)	1320 (471–2680)	55	2580 (801–4730)	72	0.016
NGAL (RFU)	223 (158–299)	55	236 (183–316)	72	ns
Osteonectin (SPARC) (RFU)	1620 (1280–1900)	55	1660 (1390–1980)	72	ns
Resistin (RFU)	6470 (5120–7680)	55	5730 (4390–7110)	72	ns
Ratio leptin/adiponectin	0.266 (0.0955–0.501)	55	0.537 (0.157–0.962)	72	0.0073

*Note:* Results compared by Mann–Whitney rank test.

Abbreviations: ACQ6, asthma control questionnaire; BAFF, B‐cell activating factor; BMI, body mass index; FeNO, fractional exhaled nitric oxide; FGF‐21, fibroblast growth factor 21; hsCRP, high‐sensitivity C‐reactive protein; IQR, interquartile range; NGAL, neutrophil gelatinase‐associated lipocalin; ns, not significant; SGRQ, St George's respiratory questionnaire; SPARC, secreted protein acidic and rich in cysteine.

### 3.2 Effect of Obesity on Adipokine Levels

**FIGURE 2:**
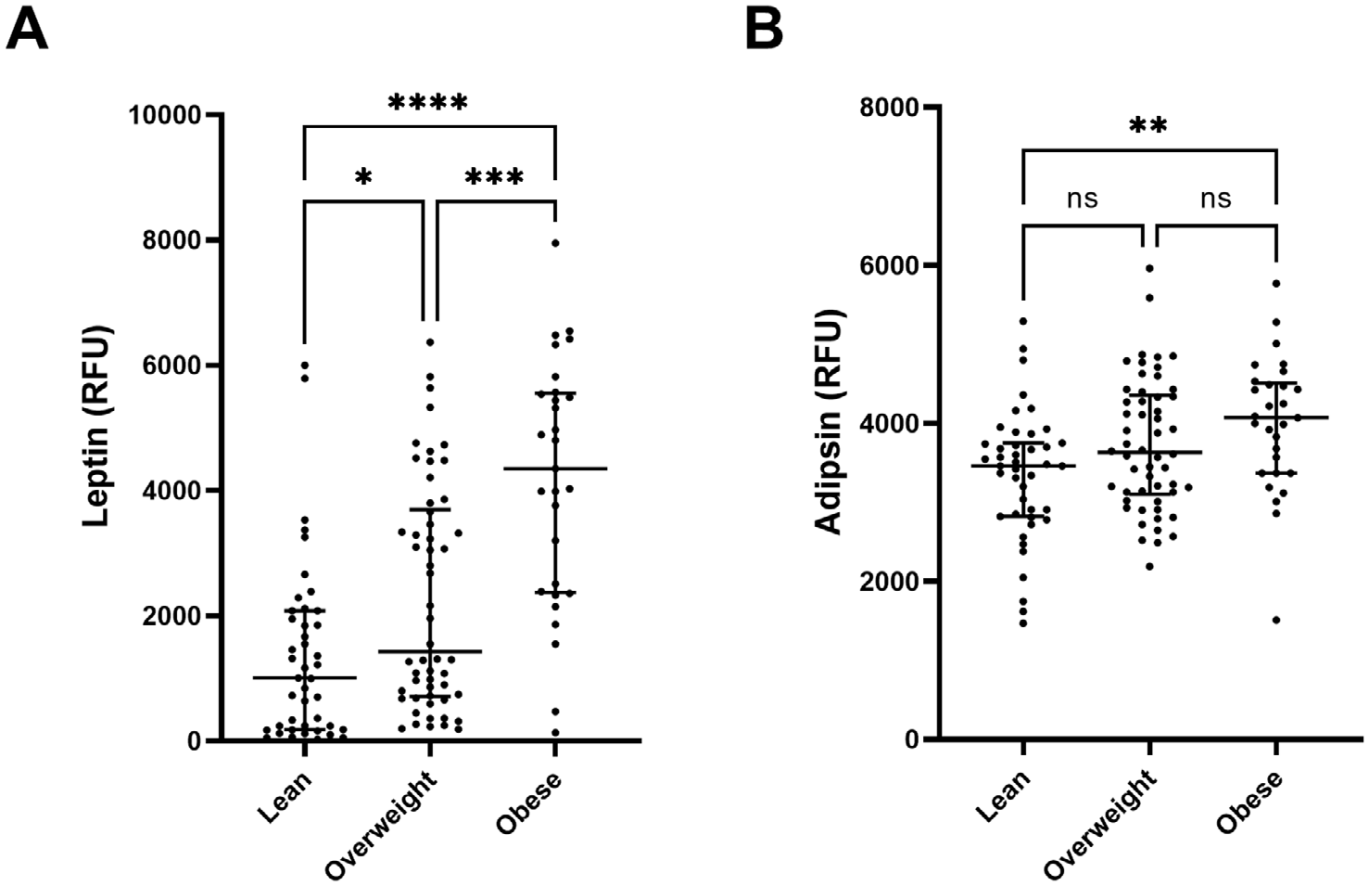
Adipokine plasma levels in patients with asthma stratified by weight status. Graphs show (A) leptin and (B) adipsin levels, presented as individual data points with median and inter‐quartile range (Kruskal–Wallis test with Dunn's correction for multiple comparisons, *p < 0.05, **p < 0.01, ***p < 0.001, and ****p < 0.0001). RFU = relative fluorescence units.

Levels of leptin and adipsin were significantly increased in the obese group compared with overweight and lean (Figure 2). No significant differences between weight status groups were found for the remainder of the measured adipokines (Table S2). However, positive correlations were observed between BMI and leptin, adipsin, and FGF‐21, and a weak negative correlation was observed between BMI and adiponectin (Table S3).

To consider the effects of sex, disease severity and age on associations between adipokines and BMI, multiple regression analyses were performed taking into account all four factors. In this case, significant associations with BMI remained for adiponectin, leptin, adipsin and NGAL (Table S4). However, none of the adipokines showed significant associations with both BMI and asthma severity.

### 3.3 Effect of Asthma Severity on Adipokine Levels

Baseline clinical characteristics and adipokine levels are shown in Table 2. As expected, MMA and SA groups differed with respect to measures of patient‐reported outcomes, lung function, and inflammation. The SA group consisted of older patients with a later onset of asthma, greater weight (BMI), poorer lung function, asthma control (ACQ6), and health status (SGRQ), as well as more blood neutrophils compared with the MMA group. Of the adipokines, chemerin and leptin were found to be significantly elevated in severe asthma compared with the milder disease group. These differences in chemerin and leptin were robust over the entire 1‐year study period (Visit 1 through Visit 6) (Table S5).

### 3.4 Effect of Sex on Adipokine Levels

**FIGURE 3:**
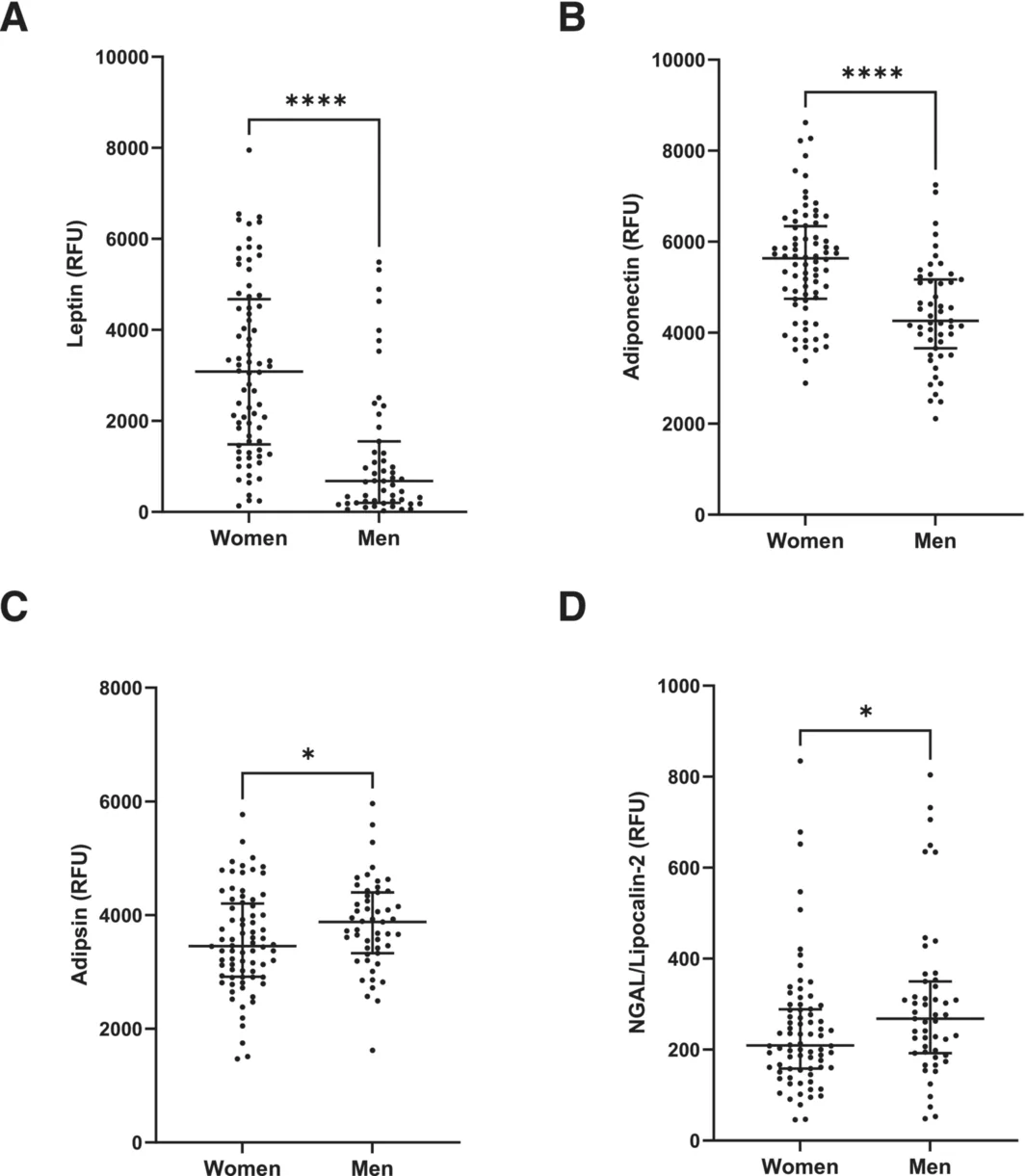
Sex differences in adipokine plasma levels for (A) leptin, (B) adiponectin, (C) adipsin, and (D) NGAL/Lipocalin‐2. Results are shown as individual data points with bars showing median and inter quartile range. (Mann–Whitney rank test, *p < 0.05 and ****p < 0.0001). RFU, relative fluorescence units.

In all patients, the levels of four adipokines were found to differ significantly between men and women. Leptin and adiponectin were both higher in female patients, whereas adipsin and NGAL were higher in males compared with females (Figure 3). Following stratification by disease group, similar patterns remained, although these did not reach significance for adipsin (Table S6). Neither BAFF, chemerin, FGF‐21, osteonectin, nor resistin were affected by sex.

In both MMA and SA groups there were more women than men (approximately 60% women in both groups, Table 1), so the association between adipokines and disease severity was also analyzed in males and females separately (Table S6). While there is a large difference in levels of leptin between women and men, an association with disease group was found in both sexes with higher levels in SA. Chemerin was also higher in SA in both women and men (but did not reach statistical significance for men, *p* = 0.069).

As the levels of both leptin and adiponectin were highly different between sexes (Figure 3), and both adipokines were associated with BMI, separate analyses of males and females were conducted. The correlation of leptin with BMI was strong in both female (Spearman *r* = 0.54, *p* < 0.0001) and male asthmatics (Spearman *r* = 0.81, *p* < 0.0001). However, the correlation of adiponectin with BMI was more pronounced in males (Spearman *r* = −0.33, *p* < 0.05) and absent in females (Spearman *r* = −0.06, ns).

### 3.5 Effect of Oral Steroids on Adipokines

A two‐week controlled intervention with oral prednisolone gave a significant reduction in hsCRP, blood eosinophils, FeNO, and periostin, and a significant increase in blood neutrophils in both patient groups (Table S7, Figure S1), confirming that the oral steroids had the expected pharmacologic effect. The lung function parameters FEV_1_ and FVC were improved in SA only and not in the MMA group. Placebo treatment gave no effect, and all parameters returned to baseline levels after recovery from prednisolone. Finally, investigating whether BMI may relate to clinical response to steroid treatment measured as change in lung function (FEV_1_ post‐steroid minus FEV_1_ pre‐steroid, ∆FEV_1_) did not reveal any significant correlation between ∆FEV_1_ and BMI (data not shown).

**FIGURE 4:**
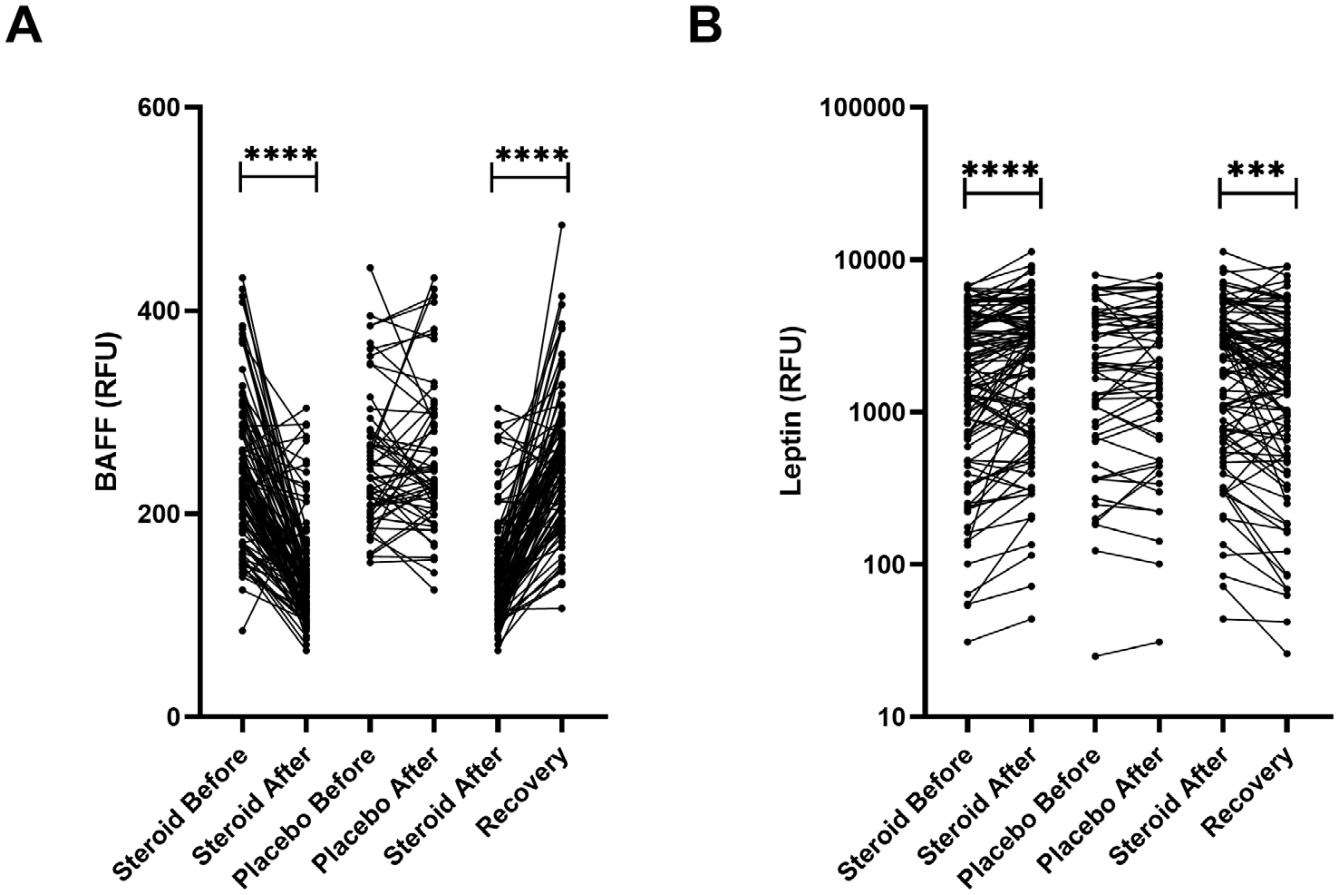
Effect of 2 weeks of oral prednisolone on (A) BAFF and (B) leptin in patients with asthma, where results are shown as paired individual data points before and after a 2‐week intervention with oral prednisolone, before and after a 2‐week placebo treatment, and from the end of the oral steroid intervention to a recovery visit, approximately 15 weeks later. Results were compared by Wilcoxon matched‐pairs signed‐rank test (***p < 0.001 and ****p < 0.0001). RFU, relative fluorescence units.

Two weeks of oral steroid treatment was found to affect most of the adipokines: adiponectin, leptin and NGAL increased, whereas adipsin, BAFF, chemerin and FGF‐21 decreased. There was however no change observed for osteonectin and resistin (Table 3). BAFF showed the largest and most consistent effect of all adipokines (Figure 4). In all instances, placebo treatment gave no effect, and all proteins affected by OCS returned to pre‐steroid levels after recovery. While both adiponectin and leptin were affected by oral steroid treatment, the effect on the ratio of leptin/adiponectin was small and non‐significant (Table 3).

**TABLE 3:** Steroid effects on adipokines in patients with mild‐to‐moderate and severe asthma.

	Asthma change (range; *n*; *p*‐value)	Mild to moderate asthma change (range; *n*; *p*‐value)	Severe asthma change (range; *n*; *p*‐value)
Adiponectin			
Baseline	5120 (*n* = 127)	5110 (*n* = 55)	5145 (*n* = 72)
Steroid	575 (108 to 1180; *n* = 106; *p* < 0.0001)	740 (360 to 1120; *n* = 50; *p* < 0.0001)	465 (−65 to 1240; *n* = 56; *p* < 0.0001)
Placebo	−200 (−670 to 170; *n* = 55; ns)	−230 (−690 to 170; *n* = 27; ns)	−150 (−590 to 298; *n* = 28; ns)
Recovery	−500 (−1060 to −240; *n* = 99; *p* < 0.0001)	−605 (−1035 to −242; *n* = 48; *p* < 0.0001)	−420 (−1090 to −210; *n* = 51; *p* < 0.0001)
Adipsin			
Baseline	3610 (*n* = 127)	3570 (*n* = 55)	3640 (*n* = 72)
Steroid	−380 (−808 to 182; *n* = 106, *p* < 0.0001)	−495 (−912 to 162; *n* = 50, *p* = 0.0001)	−290 (−748 to 258; *n* = 56; *p* = 0.024)
Placebo	−170 (−430 to 200; *n* = 55; ns)	−200 (−370 to 200; *n* = 27; ns)	−90 (−452 to 228; *n* = 28; ns)
Recovery	360 (−30.0 to 980; *n* = 99; *p* < 0.0001)	350 (−52.5 to 878; *n* = 48; *p* = 0.0003)	410 (−20 to 1090; *n* = 51; *p* < 0.0001)
BAFF			
Baseline	235 (*n* = 127)	233 (*n* = 55)	242 (*n* = 72)
Steroid	−99.5 (−142 to −47.0; *n* = 106; *p* < 0.0001)	−99.5 (−136 to −47.2; *n* = 50; *p* < 0.0001)	−96.5 (−152 to −42; *n* = 56; *p* < 0.0001)
Placebo	−2.0 (−36 to 29; *n* = 55; ns)	−8 (−36 to 17; *n* = 27; ns)	5.5 (−34.5 to 40.2; *n* = 28; ns)
Recovery	105 (60.0 to 150; *n* = 99; *p* < 0.0001)	123 (72.8 to 141; *n* = 48; *p* < 0.0001)	82 (51 to 160; *n* = 51; *p* < 0.0001)
Chemerin			
Baseline	113 (*n* = 101)	103 (*n* = 42)	120 (*n* = 59)
Steroid	−18 (−36.5 to 4.0; *n* = 49; *p* = 0.010)	−13 (−26.8 to 10.8; *n* = 28; ns)	−20 (−43 to −1; *n* = 21; *p* = 0.012)
Placebo	1.0 (−15.0 to 14.0; *n* = 30; ns)	2 (−12.5 to 14.5; *n* = 14; ns)	−3 (−25.5 to 19.2; *n* = 16; ns)
Recovery	6.0 (−7.0 to 28.0; *n* = 51; *p* = 0.020)	8.5 (−8.8 to 31.2; *n* = 26; ns)	5 (−6 to 22.5; *n* = 25; *p* = 0.058)
FGF‐21			
Baseline	140 (*n* = 127)	135 (*n* = 55)	142 (*n* = 72)
Steroid	−7.5 (−24.2 to 12.2; *n* = 106; *p* = 0.0026)	−13 (−25.2 to 5; *n* = 50; *p* = 0.0059)	−3 (−17.8 to 18.8; *n* = 56; ns)
Placebo	−3.0 (−17.0 to 18.0; *n* = 55; ns)	−3 (−18 to 22; *n* = 27; ns)	−1.5 (−15.5 to 13.8; *n* = 28; ns)
Recovery	7.0 (−8.0 to 30.0; *n* = 99; *p* = 0.0005)	9 (−5.75 to 27.8; *n* = 48; *p* = 0.0031)	7 (−13 to 31; *n* = 51; *p* = 0.039)
Leptin			
Baseline	1860 (*n* = 127)	1320 (*n* = 55)	2580 (*n* = 72)
Steroid	272 (−140 to 818; *n* = 106; *p* < 0.0001)	138 (−172 to 792; *n* = 50; *p* = 0.029)	365 (−120 to 920; *n* = 56; *p* = 0.0010)
Placebo	80 (−200 to 489; *n* = 55; ns)	90 (−140 to 440; *n* = 27; ns)	55 (−365 to 688; *n* = 28; ns)
Recovery	−234 (−1210 to 238; *n* = 99; *p* = 0.0006)	−132 (−1075 to 380; *n* = 48; ns)	−235 (−1240 to 50; *n* = 51; *p* = 0.0026)
NGAL			
Baseline	234 (*n* = 127)	223 (*n* = 55)	236 (*n* = 72)
Steroid	25 (−41.5 to 127; *n* = 106; *p* = 0.021)	34 (−37.8 to 135; *n* = 50; *p* = 0.021)	16.5 (−45.2 to 104; *n* = 56; ns)
Placebo	−21 (−55.0 to 55.5; *n* = 55; ns)	−25 (−73.5 to 30; *n* = 27; ns)	−13 (−52.2 to 56.6; *n* = 28; ns)
Recovery	−13.5 (−116 to 56.0; *n* = 99; ns)	−20.2 (−144 to 36.4; *n* = 48; *p* = 0.034)	30 (−98 to 81; *n* = 51; ns)
Osteonectin (SPARC)			
Baseline	1650 (*n* = 127)	1620 (*n* = 55)	1660 (*n* = 72)
Steroid	−5 (−255 to 210; *n* = 106; ns)	20 (−280 to 172; *n* = 50; ns)	−40 (−245 to 210; *n* = 56; ns)
Placebo	−4 (−296 to 210; *n* = 55; ns)	−4 (−330 to 260; *n* = 27; ns)	−5 (−294 to 208; *n* = 28; ns)
Recovery	82 (−150 to 360; *n* = 99; *p* = 0.050)	91 (−130 to 358; *n* = 48; ns)	30 (−260 to 370; *n* = 51; ns)
Resistin			
Baseline	5880 (*n* = 127)	6470 (*n* = 55)	5730 (*n* = 72)
Steroid	160 (−620 to 1170; *n* = 106; ns)	130 (−482 to 1115; *n* = 50; ns)	310 (−780 to 1260; *n* = 56; ns)
Placebo	−300 (−850 to 1100; *n* = 55; ns)	−570 (−1150 to 1100; *n* = 27; ns)	60 (−740 to 1380; *n* = 28; ns)
Recovery	−340 (−1530 to 390; *n* = 99; *p* = 0.0052)	−475 (−1665 to 108; *n* = 48; *p* = 0.0017)	−190 (−1430 to 520; *n* = 51; ns)
Leptin/adiponectin			
Baseline	0.387 (*n* = 127)	0.266 (*n* = 55)	0.537 (*n* = 72)
Steroid	0.006 (−0.072 to 0.098; *n* = 106; ns)	0.005 (−0.061 to 0.099; *n* = 50; ns)	0.010 (−0.089 to 0.098; *n* = 56; ns)
Placebo	0.020 (−0.014 to 0.081; *n* = 55; *p* = 0.0085)	0.019 (−0.004 to 0.070; *n* = 27; *p* = 0.019)	0.022 (−0.043 to 0.120; *n* = 28; ns)
Recovery	−0.014 (−0.135 to 0.084; *n* = 99; ns)	−0.005 (−0.124 to 0.086; *n* = 48; ns)	−0.017 (−0.136 to 0.077; *n* = 51; ns)

*Note:* Pre and post‐steroid values compared by Wilcoxon matched‐pairs signed‐rank test.

Abbreviations: BAFF, B‐cell activating factor; FGF‐21, fibroblast growth factor 21; NGAL, neutrophil gelatinase‐associated lipocalin; ns, not significant; SPARC, secreted protein acidic and rich in cysteine.

Subgroup analyses showed that there was no difference between MMA and SA groups regarding steroid‐induced changes in adiponectin, leptin, adipsin, and BAFF (Table 3). The effect of steroid on FGF‐21 and NGAL could be seen in MMA only, whereas chemerin was affected in SA only. For BAFF, the magnitude of change was greater in women than men (*p* = 0.023), particularly in the SA group (*p* = 0.0077). In the MMA group, the decrease in adipsin was larger in men (*p* = 0.051) (Table S8). Stratification by BMI showed that for all proteins, no or small differences in steroid effect existed between lean, overweight, and obese (Table S9).

Overall, there were no correlations between clinical response to steroid treatment (∆FEV_1_) and basal adipokine levels, apart from a weak yet significant positive association for resistin (Spearman *r* = 0.24, *p* = 0.01). BMI was also not associated with the magnitude of the steroid‐induced effects on adipokines, expressed as % change in their levels (data not shown).

Upon study entry, patients were taking different daily doses of inhaled corticosteroids (ICS). We examined whether ICS dose at visit 1 showed any association with adipokine levels at this time point and found that while leptin and chemerin showed weak yet significant positive correlations (Spearman *r* = 0.292, *p* < 0.001 and Spearman *r* = 0.216, *p* = 0.037 respectively), the majority of adipokines measured did not correlate with ICS dose.

### 3.6 Correlations Between Adipokine Mediators, Clinical Characteristics, Inflammation, and Patient Reported Outcomes

Overall, univariate Spearman rank correlations between adipokines and clinical and physiological parameters (Table S3) did not reveal relationships with measures of type 2 inflammation in asthma such as blood or sputum eosinophils. Circulating periostin did however weakly relate to higher adiponectin and resistin levels. On the other hand, certain adipokines were associated with potential characteristics of type 2 low asthma such as increased sputum neutrophils (osteonectin) or reduced exhaled nitric oxide (adipsin, chemerin, FGF‐21). Leptin and chemerin both correlated with higher ACQ6 scores (reduced asthma control).

Several adipokines were associated with characteristics of systemic inflammation such as increased blood neutrophils (leptin, lipocalin, resistin) or more circulating CRP (leptin, osteonectin).

Fixed airway obstruction as reflected by reduced reversibility following bronchodilator inhalation was found to be weakly associated with elevated levels of adipsin and FGF‐21. Elevated adipsin and FGF‐21 also correlated with a later onset of disease, as confirmed by a subgroup analysis of individuals with early vs. late onset disease (Table S10).

## 4 Discussion

Obesity‐related asthma is a multifactorial asthma phenotype. To test the hypothesis that mediators released from adipose tissue relate to the characteristics of asthma, circulating levels of nine selected adipokine mediators with proposed involvement in OBA were measured in BIOAIR, a well‐phenotyped cohort including mild‐to‐moderate and severe asthma patients [27, 28, 29, 30, 31]. Apart from leptin, we did not identify adipokines that were both elevated in obese patients and in those with severe compared to mild asthma. For example, we discovered that chemerin was elevated in patients with more severe asthma but, surprisingly, chemerin was not associated with obesity. However, sex, BMI, disease severity, and oral steroid use were all differentially associated with different adipokine levels, highlighting the complexity of disease pathogenesis and the importance of considering fundamental clinical characteristics when investigating possible adipose‐induced inflammation in OBA.

Patients with severe asthma had increased BMI, worse lung function, and poorer disease control compared to those with mild‐to‐moderate disease. No differences were found regarding systemic characteristics of Type 2‐high inflammation such as circulating eosinophils, periostin, and IgE. However, patients with severe asthma showed elevated blood hs‐CRP and neutrophils. This is in line with the finding that of the adipokines evaluated, patients with severe asthma also showed increased levels of both leptin and chemerin. These two pro‐inflammatory adipokines are both involved in Th17‐mediated inflammation that induces neutrophilia [32, 33]. Leptin also displays structural similarities with IL‐6 and IL‐12 and shares a similar type 1 pro‐inflammatory profile. Supporting the current findings, leptin is also associated with systemic inflammation and increased disease severity and poorer asthma control as reported by patients [11]. Accordingly, we also show that the leptin/adiponectin ratio was also significantly higher in patients with severe asthma. A higher ratio between the generally pro‐inflammatory leptin and anti‐inflammatory adiponectin is associated with poorer outcomes in relation to several cardio‐metabolic and chronic diseases, typically reflecting an adipose tissue dysfunction and a metabolically unhealthy state [34].

Our finding of increased chemerin in severe asthma confirms the findings of Zhou et al. [32], who describe a similar association in a smaller patient population and may be worth further investigation as chemerin was also relatively unaffected by other factors examined. Accordingly, when taking into account sex, BMI and disease severity in a multiple regression analysis, only chemerin remained significantly associated with disease severity but not leptin. Little is known about the role of chemerin in asthma, although it is known to act as a chemoattractant for immune cells such as dendritic cells, macrophages, and NK cells, signaling mainly through the CMKLR1 receptor (chemokine‐like receptor 1), a G protein‐coupled receptor [19]. However, studies using murine models suggest a complex and context‐dependent role for chemerin, as anti‐inflammatory effects have also been described [35].

Regarding associations between the nine adipokines and phenotypic biomarkers in asthma, few strong correlations were observed. Established measures of type 2 inflammation such as blood or sputum eosinophils, or exhaled NO, did not relate to increased adipokine levels. On the other hand, higher levels of several adipokines were associated with more blood or sputum neutrophils (leptin, lipocalin, osteonectin, resistin), more circulating CRP (leptin, osteonectin) and reduced exhaled nitric oxide (adipsin, chemerin, FGF‐21), possibly suggestive of involvement in non‐type 2 inflammatory mechanisms in asthma. Also, these characteristics are in line with chronic low‐grade systemic inflammation which is known to be associated with obesity, and also increasingly recognized as a feature present in severe asthma [36]. However, in a multiple regression analysis, simultaneously taking into account age, sex, BMI and disease severity, the lack of significant relationships with both BMI and disease severity highlights the complexity surrounding the involvement of the different adipokines in the pathobiology of severe asthma and comorbidities. The variability in the associations observed for the different adipokines also underscores the importance of addressing the roles of each of these mediators individually.

Examining the clinical effects of obesity in our investigation confirmed previous observations, in that asthmatic patients with a higher BMI also had more severe asthma with poorer lung function, worse disease control and increased systemic inflammation [1, 2, 3]. Dividing patients according to weight status into lean, overweight and obese groups was associated with strong between‐group differences for leptin and adipsin, the levels of which were increased in obese compared to lean patients, confirming earlier findings [37, 38]. Significant positive correlations with BMI were also observed for resistin, NGAL and FGF‐21, as well as an inverse association with adiponectin, which is known to be reduced in obese individuals [39].

In the current investigation we show that women have increased leptin and adiponectin, while men have increased adipsin and NGAL, all of which are produced by adipocytes. In humans and other mammals, body fat distribution differs between men and women, with women having more subcutaneous than visceral adipose tissue, an effect that has been at least partly attributed to sex hormones [40]. Subcutaneous fat produces more leptin and adiponectin than visceral fat, which could explain their well‐documented increased levels in females [41, 42], although little data exist for adipsin and NGAL. However, the extent to which sex differences in adipokine levels could contribute to sex‐related differences in asthma, such as the overrepresentation of obese women among patients with severe asthma [4], requires further investigation.

Patients with severe asthma often require maintenance oral steroid therapy [43]. Obese patients with severe asthma, however, display a reduced response to steroids due to the development of an obesity‐mediated steroid resistance [44]. Indeed, corticosteroid use was almost double per kg body weight in a general population of obese individuals compared to non‐obese ones [45]. Thus, it is essential to consider the effect of oral steroids on the outcomes of every study including severe asthma patients, especially those who are obese. BIOAIR represents an ideal cohort for this purpose as it includes a placebo‐controlled OCS intervention with prednisolone at a dosage of 0.5 mg per kg body weight for 2 weeks. The steroid intervention improved lung function in patients with severe asthma and decreased CRP, blood eosinophils, FeNO, and periostin, confirming its validity.

Oral steroids affected the majority of adipokines examined. Levels of leptin, adiponectin, and NGAL increased, while chemerin, BAFF, and FGF‐21 decreased. The effect of steroids on leptin and adiponectin levels has been reported previously [46, 47, 48]. Dexamethasone induced leptin gene expression, as well as leptin gene expression in cultured adipose tissue [46], while in a small study evaluating the effect of oral glucocorticoids on food intake, leptin increased after 2 and 7 days of 25 mg prednisolone [47]. Adiponectin also increased in healthy volunteers receiving 60 mg prednisolone/day for one week [48]. However, no studies of this size have evaluated the effect of OCS on leptin and adiponectin levels in patients with asthma. Furthermore, we found that the ratio leptin/adiponectin, which has been suggested as a marker for dysfunctional adipose tissue and metabolic syndrome‐associated cardiometabolic risk [34], remained essentially unaffected by OCS. Even less studied is the effect of OCS on BAFF in asthma. In patients with immune thrombocytopenia and increased serum BAFF, treatment with glucocorticosteroids was found to reduce BAFF, which is in line with our findings [49]. To our knowledge, the observed effects of corticosteroids on NGAL, chemerin, and FGF‐21 levels in adult patients with asthma are new and should be considered when analyzing the potential role of these adipokines in asthma or other diseases.

As the majority of adipokines were strongly affected by OCS, we considered whether high dose ICS treatment may also influence circulating adipokine levels upon study entry when patients were taking different doses of ICS. The majority of adipokines showed no relationship with ICS dose, apart from leptin and chemerin, which both showed weak positive associations. As leptin levels were increased by systemic corticosteroids, it cannot be ruled out that chronic, high‐dose ICS treatment may have contributed to elevated leptin levels. However, chemerin levels were reduced by the OCS trial, and therefore it is more likely that increased chemerin levels in patients taking higher doses of ICS are rather due to these individuals having more severe disease.

Certain adipokines were not affected by OCS use which is potentially important information when evaluating novel, disease‐relevant biomarkers. If steroids do not affect biomarker levels, then any changes may be indicative of disease‐related physiological processes rather than a consequence of the pharmacological intervention. We previously found elevated levels of osteonectin in patients with increased fluctuations in lung function, possibly due to the involvement of osteonectin in airway remodeling [25]. The finding that steroids do not affect plasma osteonectin further strengthens its potential as a disease‐related biomarker. Another adipokine, resistin, has been described to be higher in patients with severe asthma and to correlate with asthma risk [50]. Resistin was also unaffected by steroid use in the current investigation, suggesting that any changes in resistin levels may relate more to disease mechanisms than pharmacological interventions. Interestingly, resistin levels showed a weak yet significant association with clinical response to OCS which is in line with two reports both suggesting that resistin may be a biomarker of responsiveness to glucocorticoid therapy [26, 51]. However, further studies are required to examine the clinical utility of osteonectin and resistin as biomarkers because neither were strongly associated with disease severity or clinical characteristics in the current investigation.

A major strength of the current investigation is the design of the BIOAIR study which enabled us to evaluate differences between well‐characterized patients with mild‐to‐moderate and severe asthma in which we were able to reproduce known associations of BMI with lung function, inflammation, and patient reported outcomes. As the study recruited a significant number of obese male and female severe asthmatics, we could perform robust analyses of the influence of obesity and sex, as well as the effects of oral glucocorticosteroids in the 2‐week OCS intervention. Furthermore, all biomarkers were analyzed following a 4‐week treatment optimization period to reduce variability caused by differences in medication dosing. At the group level this change from an individual to a standardized treatment regime affected several parameters, demonstrating the importance of controlling for medication. In MMA, FEV_1_ and FEV_1_/FVC increased and SGRQ, ACQ6 and FeNO decreased, and in SA, adipsin and FGF‐21 decreased (Table S11). The longitudinal design also enabled us to confirm certain observed associations over time.

Limitations include the lack of a healthy control group, as well as the fact that interpreting results following several subdivisions of BIOAIR data, for example by disease group, gender, weight status and then response to OCS introduced power issues, which could perhaps be overcome in larger cohorts. Further, to obtain a deeper understanding of OBA it would have been of interest to examine adipokine levels in relation to both asthma but also comorbid features of metabolic dysfunction, such as insulin sensitivity, blood glucose or lipid levels, although such information was not available. In addition, we did not have access to local airway samples (e.g., sputum supernatants or BAL) for analysis of adipokine levels which could potentially have revealed greater associations with measures of airway inflammation. Few studies have examined airway adipokine levels, and the limited evidence available indicates that further studies are warranted before definite relationships are established. Sputum adiponectin, leptin and resistin levels have been detected from obese asthmatics [52] and a correlation between serum and sputum leptin levels has been observed [10]. Interestingly, high sputum adiponectin has also been found to predict lower odds for asthma [53].

In summary, we demonstrate several significant effects of BMI, sex and steroid treatment on circulating adipokine levels, highlighting the complexity of mechanisms underlying obesity‐related asthma and adipose‐related adipokine dysregulation. Our findings emphasize the importance of considering oral steroid therapy, which remains the most commonly used anti‐inflammatory therapy in severe asthma, despite the increasing use of newer, steroid‐sparing biological therapies, when adipokine‐related mediators are evaluated, either for understanding the underlying pathophysiology or as possible biomarkers. While body weight was, as expected, associated with marked effects on asthma severity and adipokine levels, the conclusion of our study is that none of the adipokines investigated showed strong, clear relationships with both obesity and disease severity that would be suggestive of a central role in the pathogenesis of OBA.

Thus, to disentangle the complex relationship between obesity and asthma we propose one focus of future research should be on comorbidities, to test the hypothesis that any adipokine elevations observed in severe asthma, for example, leptin, may contribute to asthma severity, at least in part, through obesity‐related comorbidities. For example, an obesity‐related asthma endotype overlapping with obstructive sleep apnea (OSA) has recently been proposed, characterized by increased exacerbations, cardiovascular risk, and difficult‐to‐treat disease [54], where leptin levels are also increased [55]. Cardiovascular disease, increasingly recognized as a comorbidity in severe asthma and strongly associated with obesity, may also represent an additional mechanistic pathway worth further examination. Population‐based evidence has revealed associations between leptin and other cardiovascular proteins with impaired lung function [56] suggestive of a potential role in both lung function decline and cardiovascular risk. Accordingly, weight‐reducing interventions such as bariatric surgery or GLP‐1 receptor agonists demonstrate profound benefits in both asthma control and cardiovascular outcomes [57, 58].

## Author Contributions

L.I.A. was responsible for adipokine measurements, data analysis and manuscript preparation together with A.J., S.‐E.D., and A.B. B.D., M.K., M.G., N.M.S., A.P., B.B., G.J., K.F.R., E.H.B., S.L.J., P.C., M.G., P.H.H., and E.N.‐M. were the clinical site leads and executed the BIOAIR study, collected patient samples and performed the clinical assessment of patients in this study. The study design was coordinated by S.‐E.D. as PI of the BIOAIR study together with the project coordinator R.M. and all clinical site leads. A.B. and A.J. were responsible for the design of the adipokine panel, performing adipokine measurements and interpretation of results. K.I. was responsible for periostin measurements. All authors contributed to the revision of the manuscript.

## Funding

The BIOAIR study was supported by The Fifth and Sixth Framework Programmes of the European Union, contract numbers: QLG1‐CT‐2000‐01185 (BIOAIR) and FOOD‐CT‐2004‐506,378 (GA2LEN) and also received unconditional support from Vitalograph. The BIOAIR study was also supported by the ChAMP (Centre for Allergy Research Highlights Asthma Markers of Phenotype) consortium which is funded by the Swedish Foundation for Strategic Research, AstraZeneca and Science for Life Laboratory Joint Research Collaboration, the Vårdal Foundation, the Swedish Heart‐Lung Foundation, the Swedish MRC, the Stockholm County Council Research Funds (ALF), the Swedish Asthma and Allergy Association's Research Foundation and Karolinska Institutet. AB also receives funding from the Konsul Th C Bergh Foundation and S.‐E.D. from the Torsten Söderberg Foundation.

## Conflicts of Interest

A.J., R.M., E.N.‐M., P.H.H., M.G., P.C., E.H.B., and L.I.A. have no conflicts of interest to disclose. B.D. reports personal fees from AstraZeneca, Teva, Sanofi and grants from Novartis and GlaxoSmithKline outside the submitted work. K.I. has received research support from Shino‐Test Co Ltd. and has a patent for the measurement of periostin issued and licensed only in Japan. B.B. reports personal fees from AstraZeneca, Boehringer Ingelheim, Menarini and GlaxoSmithKline outside the submitted work. M.G. reports grants and personal fees from Novartis, Menarini, Merck Sharp & Dohme, BMS, Galapagos, and AstraZeneca outside the submitted work. P.H.H. is currently employed by GSK but was only in academic research when the clinical study was performed. S.L.J. reports personal fees from Virtus Respiratory Research, Myelo Therapeutics GmbH, Concert Pharmaceuticals, Bayer, Synairgen, Novartis, Boehringer Ingelheim, Chiesi, Gerson Lehrman Group, resTORbio, Bioforce, Materia Medical Holdings, PrepBio Pharma, Pulmotect, Virion Health, Lallemand Pharma and AstraZeneca outside the submitted work. G.J. reports grants fand personal fees from AstraZeneca, Bayer, Sanofi, Chiesi, Eureca vzw, GlaxoSmithKline and Teva outside the submitted work. A.P. reports grants, personal fees, non‐financial support and others from GlaxoSmithKline, AstraZeneca, Boehringer Ingelheim, Chiesi, Teva, Mundipharma, Zambon, Novartis, Menarini, Sanofi/Regeneron, Roche, Fondazione Salvatore Maugeri, Chiesi and Edmond pharma outside the submitted work. K.F.R. reports grants and personal fees from AstraZeneca, Boehringer Ingelheim, Sanofi Aventis, MERCK SHARP & DOHME, Novartis, Orion Cooperation, Berlin Chemie, Roche, Chiesi and grants for research from the Ministry of Education and Science, Germany. S.‐E.D. reports personal fees from AstraZeneca, Cayman Chemicals, GlaxoSmithKline, Novartis, Regeneron, Sanofi and Teva outside the submitted work. A.B. reports institutional fees from Chiesi, GSK, and AstraZeneca and institutional grants from AstraZeneca outside the submitted work. M.K. reports personal fees from AstraZeneca, Adamed, Berlin Chemie Menarini, Chiesi, Hall Allergy, HVD, EMMA, Lek‐AM, Teva, Sanofi, Stallergenes, Novartis, GlaxoSmithKline and Zentiva outside the submitted work.

## Supporting information


**Table S1:** Adipokine mediators measured.
**Table S2:** Clinical characteristics, inflammatory mediators, and adipokines in lean, overweight, and obese patients with asthma.
**Table S3:** Spearman rank correlation analysis showing associations between adipokines with BMI, and clinical and physiological parameters. Asterisks represent the following *p*‐values, *p < 0.05, **p < 0.01, ***p < 0.001, ****p < 0.0001.
**Table S4:** Multiple regression analysis of adipokine levels in relation to age, sex, disease severity and BMI in patients with asthma.
**Table S5:** Levels of chemerin and leptin at all visits during the study.
**Table S6:** Sex differences in adipokines in patients with mild‐to‐moderate and severe asthma.
**Table S7:** Effect of oral corticosteroid treatment on clinical parameters.
**Table S8:** Sex differences in the effect of steroids on adipokines.
**Table S9:** Effect of steroid treatment on adipokines by weight status in patients with asthma.
**Table S10:** Adipokine levels in patients sub‐divided according to age of onset, early (< 12 years of age) versus late (≥ 12 years of age).
**Table S11:**. Change in adipokines, clinical and physiological parameters during the medication optimization phase Visit 1 to Visit 2.
**Figure S1:** Effect of 2 weeks of oral prednisolone on (A) blood eosinophils, (B) blood neutrophils, (C) FVC and (D) FEV_1_ in patients with asthma, where results are shown as paired individual data points (Wilcoxon matched‐pairs signed rank test, *p < 0.05, **p < 0.01, ***p < 0.001 and ****p < 0.0001).

## Data Availability

The data that support the findings of this study are available from the corresponding author upon reasonable request.
