# Supplementary material for: Adipokines in Obese Asthma: A Complex Relationship Influenced More by Sex, Weight, and Oral Steroid Treatment Than Disease Severity
Source: Allergy. 2026 Apr 3;81(9):3206–20. doi: 10.1111/all.70318 (PMC13569708; doi:10.1111/all.70318)
Supplement: Supplementary file 1 [file ALL-81-3206-s001.docx]

**Supplemental information to accompany:**

**Adipokines in obese asthma; A complex relationship influenced more by sex, weight, and oral steroid treatment than disease severity**

Lars I. Andersson, Maciej Kupczyk, Barbro Dahlén, Kenji Izuhara, Mina Gaga, Nikos M. Siafakas, Alberto Papi, Bianca Beghe, Guy Joos, Klaus F. Rabe, Elisabeth H. Bel, Sebastian L. Johnston, Pascal Chanez, Mark Gjomarkaj, Peter H. Howarth, Ewa Niżankowska-Mogilnicka, Roelinde Middelveld, Sven-Erik Dahlén, Apostolos Bossios†, and Anna James†

On behalf of the BIOAIR (Longitudinal Assessment of Clinical Course and BIOmarkers in Severe Chronic AIRway Disease) and ChAMP consortia.

†These authors contributed equally

**Contents**

**Additional Methods**

1. **BIOAIR study - Subjects, inclusion and exclusion criteria and study design**
2. **BIOAIR study - Clinical measurements**

**Tables**

**Supplementary Table S1.** Adipokine mediators measured.

**Supplementary Table S2.** Clinical characteristics, inflammatory mediators and adipokines in lean, overweight, and obese patients with asthma.

**Supplementary Table S3.** Spearman rank correlation analysis showing associations between adipokines with BMI, and clinical and physiological parameters. Asterisks represent the following p-values, *p <0.05, **p <0.01, ***p <0.001, ****p <0.0001.

**Supplementary Table S4.** Multiple regression analysis of adipokine levels in relation to age, sex, disease severity and BMI in patients with asthma.

**Supplementary Table S5.** Levels of chemerin and leptin at all visits during the study.

**Supplementary Table S6.** Sex differences in adipokines in patients with mild-to-moderate and severe asthma.

**Supplementary Table S7.** Effect of oral corticosteroid treatment on clinical parameters.

**Supplementary Table S8.** Sex differences in the effect of steroids on adipokines.

**Supplementary Table S9.** Effect of steroid treatment on adipokines by weight status in patients with asthma.

**Supplementary Table S10.** Adipokine levels in patients sub-divided according to age of onset, early (<12 years of age) vs late (≥12 years of age).

**Supplementary Table S11.** Change in adipokines, clinical and physiological parameters during the medication optimization phase Visit 1 to Visit 2.

**Figure**

**Supplementary Figure S1.** Effect of two weeks of oral prednisolone on A) eosinophils, B) neutrophils, C) FVC and D) FEV_1_ in patients with asthma, where results are shown as paired individual data points (Wilcoxon matched-pairs signed rank test, *p<0.05, **p<0.01, ***p<0.001 and ****p<0.0001).

**References**

1. **BIOAIR study - Subjects, inclusion and exclusion criteria and study design**

Asthma was diagnosed by pulmonary specialists according to published criteria [1-4]. The diagnosis of asthma had to be confirmed by at least one of the four criteria for reversible airway obstruction as documented during the last 5 years before the study or at screening visit, namely:

1) an increase in FEV_1_≥9% of predicted (or improvement of 200 ml) after administration of four puffs of 100µg salbutamol dose-aerosol inhaled via a spacer, or after additional inhalation of four puffs of 20µg ipratropium bromide administered through a large volume spacer;

2) mean diurnal variation in PEF of ≥15% on ≥4 days/week for at least 2 weeks, as calculated by the following equation: (highest PEF–lowest PEF)/mean PEF;

3) an increase in FEV_1_ of at least 400ml after a course of prednisolone 0.5mg/kg/day for 14 days;

4) in patients with a FEV_1_≥70% predicted, demonstrated bronchial hyperresponsiveness to histamine, methacholine, isocapnic hyperventilation, exercise or other indirect challenges (according to established local methods).

Subjects in the severe asthma group had been under specialist treatment for at least one year and had experienced at least one exacerbation in the past year. Exacerbations were defined either by the initiation of a course of OCS therapy in those individuals on regular ICS treatment, or for those on regular OCS therapy, a significant temporary increase in their dose of oral steroids for an acute deterioration in their disease control. Severe asthmatics had to require continuous treatment with high doses of ICS (at least 1600µg/day budesonide or beclomethasone, 800µg/day fluticasone or equivalent). For those taking oral steroids, the inhaled dose of steroids had to be at least 800µg/day budesonide or beclomethasone, 400µg/day fluticasone or equivalent. In addition, the subjects had to require continuous treatment with LABA or oral theophylline, as documented for at least one year.

Non-smokers with mild-to-moderate asthma (MMA) had stable disease and received daily treatment with a maximum of 800 µg/day budesonide or beclomethasone, 500µg/day fluticasone or equivalent. MMA subjects used SABA as needed but did not require treatment with LABA and had had no exacerbations, nor hospitalisations in the past year.

Subjects were excluded if they were pregnant, had a history of alcohol or illicit drug abuse, had other acute or chronic pulmonary disorders or had clinically relevant psychiatric disease. They were not allowed to be receiving immunosuppressants other than corticosteroids, or undergoing immunotherapy. Subjects receiving chronic oxygen therapy were also excluded.

*Study design*

Subjects were screened during a half-day visit in one of the participating clinics following which all subjects underwent a 4-week treatment optimisation period. Medication details are shown below:

MMA. Fixed moderate daily dose of inhaled steroids (800µg budesonide or beclomethasone, or 500µg fluticasone). 2. Fixed daily dose of LABA (formoterol 9-24µg b.i.d. or salmeterol 50µg b.i.d.). 3. SABA prescribed as needed. 4. No other drugs should be taken except for topical nasal drugs or treatment of comorbidity.

SA. Fixed high daily dose of inhaled steroids (1600µg budesonide or 1500µg beclomethasone, or 1000µg fluticasone). 2. Fixed daily dose of LABA (formoterol 9-24µg b.i.d. or salmeterol 50µg b.i.d.). 3. SABA prescribed as needed. 4. If applicable, dose of oral prednisolone unchanged. 5. No other drugs should be taken except for topical nasal drugs or treatment of comorbidity. (1 and 2 could be administered as a fixed combination, provided that the dosage is accurate)

The optimisation period was followed by a 2-week double-blind placebo-controlled steroid intervention, consisting of a standard course of oral prednisolone of 0.5mg/kg of body weight/day or placebo added to regular treatment. This was followed by an identical open OCS treatment of the placebo group only.

2. **BIOAIR study – Clinical measurements**

*Pulmonary function*

Lung function including forced expiratory volume in 1 second (FEV_1_), forced vital capacity (FVC), and forced inspiratory volume in 1 second (FIV_1_) were measured at each visit with daily calibrated spirometers according to previously published guidelines [5]. Patients were asked to withhold the use of short- and long-acting β-agonists for 6-10 hours before the measurements. The highest value of three technically satisfactory attempts was recorded, and predicted values were calculated according to established formulas [5]. An extended reversibility test was performed at screening visit. After FEV_1_ measurement, the patient inhaled four puffs of 100μg salbutamol (MDI via spacer) and after 15 min FEV_1_ was measured again and reversibility as percent predicted was calculated. If an improvement of 9% was not achieved, four puffs of 20μg ipratropium bromide MDI via a large volume spacer were administered, FEV_1_ was measured after 15 min and reversibility was calculated.

*Atopy and inflammatory markers*

In addition to a complete physical examination, skin prick tests to common aeroallergens were performed in all patients at the screening visit. Patients were tested with six standard allergens (Dermatophagoides pteronyssinus, mixed grasses, cat, dog, Alternaria and Aspergillus fumigatus) and two regional allergens (Southern Europe: parietaria and olive, Ferrara: parietaria and birch, Northern Europe: birch and mugwort, hazel or alder). Allergens were compared to a positive control (histamine) and a negative control (allergen diluent). ALK Soluprick (SoluprickTM, ALK lab, Copenhagen, Denmark) solutions were used and the results were expressed as a mean diameter in millimetres of two right angle measurements. A wash-out period of 3 days for short-acting and 5 days for long-acting anti-histamines was required prior to the skin prick test. A positive response was taken as a wheal ≥3 mm in comparison to the negative control. Patients were considered atopic when at least one allergen showed a positive result. Total wheal size was calculated in mm as the total of the 8 allergens.

At each visit, venous blood samples were taken for routine blood chemistry and haematology, according to the normal routine at each clinic. Total and differential white blood cell count (WBC) was measured, as well as serum C-reactive protein (CRP). Serum periostin levels were measured by ELISA using 2 rat anti-human periostin mAbs (clones SS18A and SS17B) as described previously [6].

Induced sputum was obtained at all centres according to recommendations made by the ERS (European Respiratory Society) task force on induced sputum [7]. Patients with a post-bronchodilator FEV1 % predicted <60%, a peak flow (PEF) variability of >30% during the four days preceding the sputum induction, or who had experienced severe bronchoconstriction or other adverse reactions at previous attempts, were excluded from sputum induction. The procedure was stopped if the patient displayed more than a 15% fall in FEV1 at 2 minutes after inhalation of saline (0.9% NaCl) during induction of sputum. Differential cell counts were expressed as percentage of non-squamous cells. Samples with more than 20% squamous cells or with a viability of less than 40% were excluded.

*Exhaled NO*

Exhaled nitric oxide (eNO) was measured using a NIOX analyser (Aerocrine AB, Solna, Sweden) according to ATS guidelines [8, 9] at a flow rate of exhaled air of 50 mL/second. For each patient at least three correctly executed exhalations with the device were required and the mean value was recorded.

*Serum periostin*

Serum periostin levels were measured by ELISA using 2 rat anti-human periostin mAbs (clones SS18A and SS17B) as described previously [10].

*High-sensitivity C-Reactive Protein (hsCRP)*

Serum CRP levels were measured using a standardised high sensitivity assay at the Centre for Clinical Laboratory Studies, Karolinska University Hospital, Stockholm, Sweden. Samples were analysed using a clinical Cobas c502 (8000) instrument (Roche Diagnostics, Indianapolis, USA), following a particle-enhanced immunoturbidimetric assay. No transformation of raw data was performed other than that results below LLQ were replaced with LLQ/2, which was 0.1 mg/L.

**Table S1.** Adipokine mediators measured

| **Adipokine** | **Gene name** | **Gene description/alternative names** | **Ensembl ID** | **UniProt ID** |
| --- | --- | --- | --- | --- |
| Adiponectin | ADIPOQ | C1Q and collagen domain containing | ENSG00000181092 | Q15848 |
| Adipsin | CFD | Complement factor D | ENSG00000197766 | P00746 |
| BAFF | TNFSF13B | B-cell activating factor or tumor necrosis factor ligand superfamily member 13B | ENSG00000102524 | Q9Y275 |
| Chemerin | RARRES2 | retinoic acid receptor responder protein 2 | ENSG00000106538 | Q99969 |
| FGF-21 | FGF21 | Fibroblast growth factor 21 | ENSG00000105550 | Q9NSA1 |
| Leptin | LEP | Leptin | ENSG00000174697 | P41159 |
| NGAL | LCN2 | Neutrophil gelatinase-associated lipocalin (NGAL) | ENSG00000148346 | P80188 |
| Osteonectin | SPARC | Secreted protein acidic and rich in cysteine (SPARC) | ENSG00000113140 | P09486 |
| Resistin | RETN | Adipose tissue-specific secretory factor (ADSF) | ENSG00000104918 | Q9HD89 |

**Supplementary Table S2.** Clinical characteristics, inflammatory mediators and adipokines in lean, overweight, and obese patients with asthma.

|  | **Lean** | **N** | **Overweight (OW)** | **N** | **Obese** | **N** | **Between-group comparisons** | | |
| --- | --- | --- | --- | --- | --- | --- | --- | --- | --- |
|  | **Median (IQR)** |  | **Median (IQR)** |  | **Median (IQR)** |  | **Lean vs OW** | **Lean vs Obese** | **OW vs Obese** |
| FVC (% predicted) | 104.5 (91.4–114) | 41 | 98.2 (89.0–114) | 54 | 89.4 (80.0–101.0) | 29 | ns | 0.010 | 0.022 |
| FEV_1_/FVC | 0.71 (0.64–0.82) | 41 | 0.74 (0.64–0.80) | 54 | 0.67 (0.60–0.74) | 29 | ns | ns | ns |
| FEV_1_ (% predicted) | 90.5 (76.0–102.7) | 41 | 86.6 (73.4–101.1) | 54 | 70.9 (60.6–83.8) | 29 | ns | 0.0004 | 0.0023 |
| ACQ6 (score) | 0.58 (0.042–1.29) | 40 | 1.17 (0.33–2.0) | 53 | 1.17 (0.58–2.33) | 29 | ns | 0.024 | ns |
| SGRQ (score) | 20.2 (9.2–40.0) | 33 | 37.5 (16.8–48.1) | 47 | 43.2 (23.8–58.8) | 29 | ns | 0.014 | ns |
| Blood eosinophils (cells/µL) | 250 (125–340) | 41 | 280 (140–408) | 52 | 200 (110–390) | 28 | ns | ns | ns |
| Blood neutrophils (cells/µL) | 3690 (3140–4680) | 41 | 3680 (3060–4900) | 52 | 5100 (4220–6610) | 28 | ns | 0.0018 | 0.0017 |
| hsCRP (mg/L) | 0.64 (0.34-1.2) | 33 | 1.8 (0.66–2.9) | 46 | 5.4 (2.4–11.2) | 24 | 0.014 | <0.0001 | 0.0005 |
| FeNO (ppb) | 24.3 (15.8–39.9) | 23 | 25.3 (15.2–40.5) | 31 | 40.7 (7.2–79.2) | 8 | ns | ns | ns |
| Sputum Neutrophils (%) | 36.6 (14.5–65.7) | 26 | 44.8 (23.5–71.8) | 36 | 40.2 (23.1–56.4) | 22 | ns | ns | ns |
| Sputum Eosinophils (%) | 1.0 (0.0–3.1) | 26 | 2.5 (0.6-13.7) | 36 | 2.4 (0.7–15.3) | 22 | ns | ns | ns |
| Periostin (ng/ml) | 77 (64–86) | 39 | 78 (63-102) | 52 | 80 (68–91) | 26 | ns | ns | ns |
| Adiponectin (RFU) | 5390 (4710–6070) | 43 | 4600 (3910-5760) | 54 | 5120 (4040–5900) | 29 | ns | ns | ns |
| Adipsin (RFU) | 3460 (2820–3750) | 43 | 3630 (3100-4360) | 54 | 4070 (3370–4510) | 29 | ns | 0.0044 | ns |
| BAFF (RFU) | 230 (192–263) | 43 | 232 (198-276) | 54 | 270 (186–306) | 29 | ns | ns | ns |
| Chemerin (RFU) | 114 (102–142) | 33 | 115 (94-160) | 44 | 103 (82–146) | 23 | ns | ns | ns |
| FGF-21 (RFU) | 133 (117–150) | 43 | 142 (121-173) | 54 | 150 (129–174) | 23 | ns | ns (0.063) | ns |
| Leptin (RFU) | 1010 (188–2080) | 43 | 1430 (711-3700) | 54 | 4350 (2380–5560) | 29 | 0.029 | <0.0001 | 0.0009 |
| NGAL (Lipocalin-2) (RFU) | 225 (161–299) | 43 | 220 (152-316) | 54 | 282 (203–352) | 29 | ns | ns | ns |
| Osteonectin (SPARC) (RFU) | 1600 (1300–1920) | 43 | 1660 (1360-1870) | 54 | 1720 (1420–2170) | 29 | ns | ns | ns |
| Resistin (RFU) | 5850 (4760–7150) | 43 | 5760 (4340-7280) | 54 | 6180 (5450–8010) | 29 | ns | ns | ns |
| Leptin/Adiponectin | 0.201 (0.050-0.399) | 43 | 0.378 (0.167-0.761) | 54 | 0.843 (0.520-1.25) | 29 | 0.0072 | <0.0001 | 0.0010 |
| Resistin/Adiponectin | 1.15 (0.811-1.40) | 43 | 1.25 (0.774-1.94) | 54 | 1.42 (1.008-1.88) | 29 | ns | 0.045 | ns |

Abbreviations: OW, overweight; IQR, interquartile range; ns, not significant; BMI, body mass index; ACQ6, asthma control questionnaire; SGRQ, St George's respiratory questionnaire; hsCRP, high-sensitivity C-reactive protein; FeNO, fractional exhaled nitric oxide; BAFF, B-cell activating factor; FGF‑21, fibroblast growth factor 21; NGAL, neutrophil gelatinase-associated lipocalin; SPARC, secreted protein acidic and rich in cysteine.

**Supplementary Table S3.** Spearman rank correlation analysis showing associations between adipokines with BMI, and clinical and physiological parameters. Spearman rank correlation coefficients (Spearman r) are shown and asterisks represent the following p-values, *p<0.05, **p<0.01, ***p<0.001, ****p<0.0001.

|  | Adiponectin | Adipsin | BAFF | Chemerin | FGF-21 | Leptin | NGAL | Osteonectin | Resistin | Leptin/ Adiponectin | Resistin/ Adiponectin | BMI |
| --- | --- | --- | --- | --- | --- | --- | --- | --- | --- | --- | --- | --- |
| Blood Eosinophils | -0.07 | 0.04 | 0.12 | 0.13 | 0.06 | -0.10 | 0.04 | 0.10 | 0.16 | -0.10 | 0.12 | -0.11 |
| Blood Neutrophils | -0.09 | -0.00 | -0.11 | 0.16 | 0.03 | 0.24** | 0.37**** | -0.11 | 0.24** | 0.28** | 0.22* | 0.27** |
| Sputum Neutrophils | -0.10 | 0.02 | -0.07 | -0.06 | -0.02 | -0.17 | -0.19 | 0.23* | -0.02 | -0.11 | 0.01 | 0.04 |
| Sputum Eosinophils | -0.14 | -0.10 | -0.08 | 0.05 | -0.04 | 0.03 | 0.14 | 0.15 | 0.13 | -0.05 | 0.16 | 0.18 |
| hsCRP | -0.14 | 0.16 | 0.22 | 0.17 | 0.09 | 0.47**** | 0.19 | 0.20* | 0.15 | 0.51**** | 0.24* | 0.57**** |
| Periostin | 0.27** | 0.13 | 0.06 | 0.04 | 0.15 | -0.01 | 0.05 | 0.08 | 0.21* | -0.09 | -0.04 | -0.01 |
| FeNO | -0.05 | -0.25* | -0.16 | -0.31* | -0.28* | -0.09 | 0.11 | -0.06 | 0.06 | -0.07 | 0.07 | -0.06 |
| FEV_1_% | 0.10 | -0.08 | -0.09 | -0.15 | -0.08 | -0.10 | -0.11 | -0.16 | -0.12 | -0.12 | -0.14 | -0.33*** |
| FVC% | 0.20* | -0.08 | -0.04 | -0.04 | -0.05 | -0.03 | -0.10 | 0.03 | -0.10 | -0.08 | -0.18* | -0.26** |
| ACQ6 | 0.05 | -0.07 | 0.06 | 0.23* | -0.03 | 0.29** | 0.05 | 0.08 | -0.01 | 0.28** | -0.02 | 0.31*** |
| Age of onset | 0.06 | 0.22* | 0.03 | 0.13 | 0.21* | 0.03 | -0.09 | 0.15 | 0.01 | -0.03 | 0.01 | 0.15 |
| Reversibility | -0.06 | -0.25** | -0.02 | -0.08 | -0.24** | -0.17 | -0.14 | -0.01 | -0.03 | 0.16 | -0.04 | -0.11 |
| BMI | -0.18* | 0.28** | 0.10 | -0.03 | 0.20* | 0.53**** | 0.17 | 0.11 | 0.13 | 0.57**** | 0.23* |  |

Abbreviations: BAFF, B-cell activating factor; FGF‑21, fibroblast growth factor 21; NGAL, neutrophil gelatinase-associated lipocalin; BMI, body mass index; hsCRP, high-sensitivity C-reactive protein; FeNO, fractional exhaled nitric oxide; ACQ6, asthma control questionnaire.

**Supplementary Table S4.** Multiple regression analysis of adipokine levels in relation to age, sex, disease severity and BMI in patients with asthma.

|  | **Age** | **Sex** | **BMI** | **Disease severity** |  |
| --- | --- | --- | --- | --- | --- |
| Adiponectin | 0.13 (-0.01 - 0.28) | 0.10 (0.06 - 0.14)* | -0.37 (-0.63 - -0.11)* | 0.005 (-0.04 - 0.05) |  |
| Leptin | -0.06 (-0.57 - 0.46) | 0.64 (0.51 - 0.77)* | 3.90 (2.98 - 4.84)* | -0.005 (-0.15 - 0.14) |  |
| Adipsin | 0.15 (0.00 - 0.31)* | -0.04 (-0.08 - -0.00)* | 0.30 (0.03 - 0.58)* | -0.01 (-0.06 - 0.03) |  |
| BAFF | -0.05 (-0.23 - 0.12) | 0.03 (-0.02 - 0.07) | 0.07 (-0.24 - 0.39) | 0.008 (-0.04 - 0.06) |  |
| Chemerin | 0.11 (-0.13 - 0.35) | 0.01 (-0.06 - 0.07) | -0.16 (-0.63 - 0.30) | 0.08 (0.01 - 0.15)* |  |
| Resistin | 0.00 (-0.26 - 0.26) | 0.00 (-0.06 - 0.07) | 0.49 (0.28 - 0.96)* | -0.07 (-0.14 - 0.00) |  |
| Osteonectin (SPARC) | 0.11 (-0.05 - 0.26) | -0.01 (-0.05 - 0.03) | 0.06 (-0.22 - 0.35) | 0.03 (-0.02 - 0.07) |  |
| FGF-21 | 0.14 (0.01 - 0.27)* | -0.02 (-0.05 - 0.01) | 0.20 (-0.03 - 0.44) | -0.01 (-0.05 – 0.03) |  |
| NGAL (Lipocalin-2) | -0.29 (-0.60 – 0.05) | -0.08 (-0.16 - 0.01) | 0.70 (0.09 - 1.30)* | 0.02 (-0.08 – 0.11) |  |

Abbreviations: BMI, body mass index; BAFF, B-cell activating factor; SPARC, secreted protein acidic and rich in cysteine; FGF‑21, fibroblast growth factor 21; NGAL, neutrophil gelatinase-associated lipocalin. Results presented as coefficient (95% confidence interval), *p<0.05

**Supplementary Table S5**. Levels of chemerin and leptin at all visits during the study

|  | **Mild to Moderate Asthma (MMA)** | **N** | **Severe asthma (SA)** | **N** | **Between group comparisons** |
| --- | --- | --- | --- | --- | --- |
|  | **Median (IQR)** |  | **Median (IQR)** |  | **MMA vs SA** |
| Chemerin |  |  |  |  |  |
| Visit 1 | 102 (81.2–136) | 44 | 127 (90.0–161) | 51 | ns (0.079) |
| Visit 2 | 103 (90.8–124) | 42 | 120 (101-162) | 59 | 0.0053 |
| Visit pre-OCS | 103 (84.5–130) | 37 | 119 (97.5-154) | 49 | ns (0.060) |
| Visit post-OCS | 96.0 (68.2–120) | 32 | 108 (97.0–125) | 32 | ns |
| Visit 4 | 105 (81.8-131) | 34 | 112 (98.5-151) | 42 | ns |
| Visit 5 | 102 (77.2-140) | 28 | 143 (102-177) | 37 | 0.018 |
| Visit 6 | 104 (81.5–130) | 29 | 134 (110-188) | 31 | 0.0025 |
| Leptin |  |  |  |  |  |
| Visit 1 | 1420 (474-2400) | 56 | 2280 (1030-4640) | 72 | 0.0018 |
| Visit 2 | 1320 (471-2680) | 55 | 2580 (801-4730) | 72 | 0.016 |
| Visit pre-OCS | 1530 (472-2880) | 53 | 2740 (712-4730) | 68 | 0.034 |
| Visit post-OCS | 2060 (595-3240) | 52 | 3200 (1080-5270) | 64 | 0.0094 |
| Visit 4 | 1520 (471-2880) | 52 | 1750 (627-3680) | 65 | ns |
| Visit 5 | 1400 (525-2760) | 43 | 1960 (1130-4640) | 56 | ns (0.052) |
| Visit 6 | 1500 (473-2930) | 40 | 2940 (1080-4840) | 48 | 0.0097 |

Abbreviations: IQR, interquartile range; OCS, oral corticosteroid; ns, not significant.

**Supplementary Table S6.** Sex differences in adipokines in patients with mild-to-moderate and severe asthma.

|  | **Mild to Moderate Asthma (MMA)** | **N** | **Severe asthma (SA)** | **N** | **Between group comparisons** |
| --- | --- | --- | --- | --- | --- |
|  | **Median (IQR)** |  | **Median (IQR)** |  | **MMA vs SA** |
| Adiponectin All | 5110 (4200–5860) | 55 | 5145 (3970–5930) | 72 | ns |
| Women | 5670 (4890–6140) **** | 34 | 5560 (4450-6470) ** | 42 | ns |
| Men | 4520 (3500–5100) **** | 21 | 4240 (3930-5310) ** | 30 | ns |
| Adipsin All | 3570 (3010–4160) | 55 | 3640 (3140–4400) | 72 | ns |
| Women | 3420 (2910-4270) | 34 | 3510 (3070-4140) | 42 | ns |
| Men | 3740 (3360-4110) | 21 | 3900 (3300-4440) | 30 | ns |
| BAFF All | 233 (208–268) | 55 | 242 (193-280) | 72 | ns |
| Women | 234 (199-271) | 34 | 252 (195-309) | 42 | ns |
| Men | 230 (208-251) | 21 | 224 (185-273) | 30 | ns |
| Chemerin All | 103 (90.8–124) | 42 | 120 (101-162) | 59 | **0.0053** |
| Women | 105 (86.0–124) | 26 | 132 (100-162) | 35 | **0.039** |
| Men | 100 (91.5–127) | 16 | 116 (101-177) | 24 | ns |
| FGF-21 All | 135 (117–170) | 55 | 142 (123-164) | 72 | ns |
| Women | 133 (117–168) | 34 | 142 (119-155) | 42 | ns |
| Men | 137 (122–171) | 21 | 143 (130-172) | 30 | ns |
| Leptin All | 1320 (471–2680) | 55 | 2580 (801-4730) | 72 | **0.016** |
| Women | 2020 (1320-3440) **** | 34 | 3630 (2140-5460) **** | 42 | **0.014** |
| Men | 363 (180-854) **** | 21 | 976 (244-2420) **** | 30 | **0.039** |
| NGAL All | 223 (158-299) | 55 | 236 (183-316) | 72 | ns |
| Women | 197 (125-267) ** | 34 | 235 (181-301) | 42 | ns |
| Men | 268 (201-398) ** | 21 | 258 (182-342) | 30 | ns |
| Osteonectin All | 1620 (1280-1900) | 55 | 1660 (1390-1980) | 72 | ns |
| Women | 1630 (1220-1900) | 34 | 1730 (1380-1970) | 42 | ns |
| Men | 1620 (1320-1940) | 21 | 1600 (1380-2110) | 30 | ns |
| Resistin All | 6470 (5120-7680) | 55 | 5730 (4390-7110) | 72 | ns |
| Women | 5880 (5040-7590) | 34 | 5660 (4440-7130) | 42 | ns |
| Men | 6810 (5780-8060) | 21 | 5820 (4320-7120) | 30 | ns |
| Leptin/Adipon. All | 0.266 (0.0955-0.501) | 55 | 0.537 (0.157-0.962) | 72 | **0.0073** |
| Women | 0.359 (0.218-0.676) **** | 34 | 0.745 (0.439-1.015) ** | 42 | **0.0064** |
| Men | 0.0860 (0.0422-0.259) **** | 21 | 0.224 (0.0612-0.645)** | 30 | ns |
| Resistin/Adipon. All | 1.29 (0.932-1.71) | 55 | 1.19 (0.805-1.61) | 72 | ns |
| Women | 1.026 (0.872-1.48) *** | 34 | 1.14 (0.765-1.47) | 42 | ns |
| Men | 1.64 (1.26-1.98) *** | 21 | 1.31 (0.986-2.03) | 30 | ns |

Abbreviations: IQR, interquartile range; ns, not significant; BAFF, B-cell activating factor; FGF‑21, fibroblast growth factor 21; NGAL, neutrophil gelatinase-associated lipocalin; SPARC, secreted protein acidic and rich in cysteine.

**Supplementary Table S7.** Effect of oral corticosteroid treatment on clinical parameters.

|  | **Mild Asthma, Change (range; N; p-value)** | **Severe Asthma, Change (range; N; p-value)** |
| --- | --- | --- |
| **FVC (% predicted)** |  |  |
| Baseline | 106 (n = 53) | 92 (n = 72) |
| Steroid | 1.7 (-3.5 – 5.4; n = 46; ns) | 2.9 (-3.3 – 10.8; n = 69; p = 0.010) |
| Placebo | 0.5 (-3.3 – 3.7; n = 26; ns) | -0.6 (-6.9 – 6.2; n = 32; ns) |
| Recovery | -1.2 (-7.8 – 3.2; n = 48; p = 0.046) | -3.9 (-13.4 – 2.9; n = 65; p = 0.0029) |
| **FEV_1_/FVC** |  |  |
| Baseline | 0.74 (n = 53) | 0.69 (n = 72) |
| Steroid | 0.00 (-0.03 – 0.04; n = 46; ns) | 0.02 (-0.02 – 0.06; n = 69; p = 0.014) |
| Placebo | -0.02 (-0.03 – 0.02; n = 26; ns) | -0.02 (-0.06 – 0.03; n = 32; ns) |
| Recovery | 0.00 (-0.03 – 0.02; n = 48; ns) | -0.01 (-0.05 – 0.02; n = 65; ns) |
| **FEV_1_ (% predicted)** |  |  |
| Baseline | 94 (n = 53) | 76 (n = 72) |
| Steroid | 0.4 (-3.7 – 5.2; n = 47; ns) | 3.3 (-2.2 – 11.1; n = 69; p = 0.0009) |
| Placebo | -1.1 (-3.8 – 1.5; n = 27; ns) | -2.2 (-6.6 – 4.2; n = 32; ns) |
| Recovery | -1.9 (-8.3 – 2.0; n = 48; p = 0.017) | -2.9 (-13.2 – 2.2; n = 65; p = 0.0023) |
| **ACQ6** |  |  |
| Baseline | 0.33 (n = 54) | 1.67 (n = 69) |
| Steroid | 0.0 (-0.17 – 0.16; n = 51; ns) | -0.16 (-0.67 – 0.33; n = 66; ns) |
| Placebo | 0.0 (-0.33 – 0.13; n = 28; ns) | -0.08 (-0.54 – 0.33; n = 30; ns) |
| Recovery | 0.0 (-0.17 – 0.33; n = 51; ns) | 0.16 (-0.25 – 0.67; n = 61; ns) |
| **SGRQ** |  |  |
| Baseline | 16.8 (n = 45) | 44.0 (n = 65) |
| Steroid | -0.2 (-4.3 – 4.2; n = 16; ns) | -1.2 (-4.8 – 5.4; n = 32; ns) |
| Placebo | -1.4 (-4.6 – 0.8; n = 24; ns) | -2.1 (-5.2 – 4.3; n = 27; ns) |
| Recovery | -2.2 (-9.4 – 1.0; n = 18; ns) | 1.1 (-6.2 – 7.8; n = 27; ns) |
| **Blood eosinophils** |  |  |
| Baseline | 250 (n = 53) | 250 (n = 69) |
| Steroid | -170 (-265 – -35; n = 49; p = <0.0001) | -100 (-340 – -20; n= 61; p =<0.0001) |
| Placebo | 10 (-40 – 100; n = 27; ns) | 0 (-30 – 120; n = 28; ns) |
| Recovery | 140 (1 0 – 240; n = 47; p = <0.0001) | 100 (20 – 300; n = 58; p = <0.0001) |
| **Blood neutrophils** |  |  |
| Baseline | 3560 (n = 53) | 4750 (n = 70) |
| Steroid | 3560 (1380 – 5700; n= 49; p= <0.0001) | 2570 (760 – 4580); n= 61; p = <0.0001) |
| Placebo | 300 (-460 – 900; n = 27; ns) | -190 (-680 – 210; n = 28; ns) |
| Recovery | -3540 (-6370 – -1400; n = 59; p=<0.0001) | -2680 (-4800 – -740; n=58; p=<0.0001) |
| **hsCRP** |  |  |
| Baseline | 1.1 (n = 47) | 2.3 (n = 57) |
| Steroid | -0.5 (-2.1 – 0.0; n = 47; p = 0.0003) | -0.4 (-1.8 – 0.0; n = 56; p = 0.0012) |
| Placebo | 0.0 (-0.8 – 0.6; n = 27; ns) | -0.2 (-1.6 – 1.0; n = 24; ns) |
| **FeNO** |  |  |
| Baseline | 22 (n = 28) | 30 (n = 34) |
| Steroid | -4.8 (-14.8 – 3.6; n = 23; p = 0.023) | -8.4 (-27.4 – -2.0; n = 26; p = <0.0001) |
| Placebo | -2.0 (-5.2 – 0.3; n = 18; p = 0.043) | -5.1 (-16.9 – 2.2; n = 12; ns) |
| Recovery | 6.4 (-4.5 – 24.0; n = 23; ns) | 5.3 (-1.4 – 48.2; n = 23; p = 0.046) |
| **Periostin** |  |  |
| Baseline | 75 (n = 50) | 79 (n = 68) |
| Steroid | -12 (-24 - -6; n = 46; p = <0.0001) | -13 (-30 – 0; n = 57; p = <0.0001) |
| Placebo | 0 (-6 – 10; n = 26; ns) | 2 (-10 – 10; n = 28; ns) |
| Recovery | 13 (2 – 24; n = 44; p = <0.0001) | 15 (0 – 28; n = 47; p = <0.0001) |
| **Leptin/Adiponectin** |  |  |
| Baseline | 0.266 (n=55) | 0.537 (n=72) |
| Steroid | 0.005 (-0.061 – 0.099; n=50; ns) | 0.010 (-0.089 – 0.098; n=56; ns) |
| Placebo | 0.019 (-0.004 – 0.070; n=27; p=0.019) | 0.022 (-0.043 – 0.120; n=28; ns) |
| Recovery | -0.005 (-0.124 – 0.086; n= 48; ns) | -0.017 (-0.136 – 0.077; n=51; ns) |
| **Resistin/Adiponectin** |  |  |
| Baseline | 1.29 (n=55) | 1.19 (n=72) |
| Steroid | -0.101 (-0.312– 0.052; n=50; p=0.018) | -0.125(-0.347–0.075; n=56; p=0.0070) |
| Placebo | -0.083 (-0.214 – 0.133; n=27; ns) | 0.006 (-0.095 – 0.301; n=28; ns) |
| Recovery | 0.073 (-0.197 – 0.212; n=48; ns) | 0.106 (-0.094 – 0.376; n=51; p=0.021) |

Abbreviations: ns, not significant; ACQ6, asthma control questionnaire; SGRQ, St George's respiratory questionnaire; hsCRP, high-sensitivity C-reactive protein; FeNO, fractional exhaled nitric oxide.

**Supplementary Table S8.** Sex differences in the effect of steroids on adipokines.

| **Analyte** | **Mild to Moderate Asthma**  **Change (range; N; p-value)** | **Severe Asthma**  **Change (range; N; p-value)** |
| --- | --- | --- |
| **Adiponectin** |  |  |
| All | **740** (360 - 1120; n = 50; p = <0.0001) | **465** (-65 - 1240; n = 56; p = <0.0001) |
| Women | **540** (20 – 1220; n = 31; p = <0.0001) | **430** (-65 – 1220; n = 36; p = 0.0014) |
| Men | **800** (530 – 1100; n = 19; p = <0.0001) | **610** (-30 – 1300; n = 20; p = 0.0049) |
| **Adipsin** |  |  |
| All | **-495** (-912 – 162; n = 50, p = 0.0001) | **-290** (-748 – 258; n = 56; p = 0.0242) |
| Women | **-250** (-740 – 230; n = 31; ns) | **-140** (-812 – 258; n = 36; ns) |
| Men | **-740** (-1090 - -450; n= 19; p = <0.0001) | **-410** (-680 – 328; = 20; ns) |
| **BAFF** |  |  |
| All | **-99.5** (-136 – -47.2; n= 50; p= <0.0001) | **-96.5** (-152 - -42; n = 56; p = <0.0001) |
| Women | **-103** (-143 – -50; n = 31; p = <0.0001) | **-114** (-162 – -69; n = 36; p = <0.0001) |
| Men | **-97** (-134 – -27; n = 19; p = <0.0001) | **-46.5** (-105 – -32.5; n = 20; p = 0.0005) |
| **FGF-21** |  |  |
| All | **-13** (-25.2 – 5; n = 50; p = 0.0059) | **-3** (-17.8 – 18.8; n = 56; ns) |
| Women | **-7** (-24 – 15; n = 31; ns) | **-5.5** (-24.8 – 14.5; n = 36; ns) |
| Men | **-18** (-44 – -12; n = 19; p = <0.0001) | **3.5** (-12.5 – 25.2; n = 20; ns) |
| **Leptin** |  |  |
| All | **138** (-172 – 792; n = 50; p = 0.0290) | **365** (-120 – 920; n = 56; p = 0.0010) |
| Women | **320** (-450 – 1080; n = 31; ns) | **405** (-120 – 1700; n = 36; p = 0.0016) |
| Men | **129** (13 – 406; n = 19; p = 0.0361) | **122** (-110 – 686; n = 20; ns) |
| **NGAL** |  |  |
| All | **34** (-37.8 – 135; n = 50; p = 0.0211) | **16.5** (-45.2 – 104; n = 56; ns) |
| Women | **71** (-20 – 134; n = 31; p = 0.0164) | **16.5** (-52 – 103; n = 36; ns) |
| Men | **22.5** (-65 – 167; n = 19; ns) | **15** (-32.4 – 122; n = 20; ns) |
| **Osteonectin (SPARC)** |  |  |
| All | **20** (-280 – 172; n = 50; ns) | **-40** (-245 – 210; n = 56; ns) |
| Women | **59** (-150 – 340; n = 31; ns) | **-25** (-225 – 225; n = 36; ns) |
| Men | **-30** (-310 – 110; n = 19; ns) | **-60** (-340 – 205; n = 20; ns) |
| **Resistin** |  |  |
| All | **130** (-482 – 1115; n = 50; ns) | **310** (-780 – 1260; n = 56; ns) |
| Women | **310** (-250 – 1190; n = 31; ns) | **530** (-440 – 1360; n = 36; p = 0.0604) |
| Men | **110** (-510 – 1050; n = 19; ns) | **-325** (-1333 – 506; n = 20; ns) |
| **Chemerin** |  |  |
| All | **-13** (-26.8 – 10.8; n = 28; ns) | **-20** (-43 – -1; n= 21; p = 0.012) |
| Women | **-15** (-26 – 11; n = 15; ns) | **-24.5** (-43.2 – -1; n = 14; p = 0.0063) |
| Men | **-8** (-40 – 17.5; n = 13; ns) | **-17** (-43 – 26; n = 7; ns) |
| **Leptin/Adiponectin** |  |  |
| All | **0.005** (-0.061 – 0.099; n = 50; ns) | **0.010** (-0.089 – 0.098; n = 56; ns) |
| Women | **0.006** (-0.063 – 0.110; n = 31; ns) | **0.017** (-0.10 – 0.130; n = 36; ns) |
| Men | **0.004** (-0.019 – 0.050; n = 19; ns) | **0.008** (-0.076 – 0.052; n = 20; ns) |
| **Resistin/Adiponectin** |  |  |
| All | **-0.101** (-0.312– 0.052; n = 50; p = 0.018) | **-0.125** (-0.347 – 0.075;n=56;p=0.0070) |
| Women | **-0.043** (-0.150 – 0.125; n = 31; ns) | **-0.022** (-0.231 – 0.126; n = 36; ns) |
| Men | **-0.246** (-0.394 – -0.091; n=19; p = 0.011) | **-0.242**(-0.478 – -0.029;n=20;p=0.0056) |

Abbreviations: ns, not significant; BAFF, B-cell activating factor; FGF‑21, fibroblast growth factor 21; NGAL, neutrophil gelatinase-associated lipocalin; SPARC, secreted protein acidic and rich in cysteine.

**Supplementary Table S9.** Effect of steroid treatment on adipokines by weight status in patients with asthma.

|  | **Lean**  **Change (range; N; p-value)** | **Overweight**  **Change (range; N; p-value)** | **Obese**  **Change (range; N; p-value)** |
| --- | --- | --- | --- |
| **Leptin** | 155 (-80-840; n=35; **p=0.012)** | 225 (-219-644; n=47; ns) | 530 (0-2070; n=23; **p=0.0068)** |
| **Adiponectin** | 420 (-160-1170; n=35; **p=0.0008)** | 630 (370-1090; n=47; **p<0.0001)** | 660 (360-1650; n=23; **p=0.0003)** |
| **Adipsin** | -290 (-840-110; n=35; **p=0.0015)** | -330 (-800-230; n=47; **p=0.010)** | -470 (-760-320; n=23; ns) |
| **BAFF** | -92 (-130--32; n=35; **p<0.0001)** | -106 (-134--39; n=47; **p<0.0001)** | -127 (-184--62; n=23; **p<0.0001)** |
| **Chemerin** | -11 (-27 – 10; n=19; ns) | -22 (-43 – -3.2; n=18; **p=0.010**) | -22 (-36 – 12; n=10; ns) |
| **FGF-21** | -9 (-25-5; n=35; ns) | -8 (-23-12; n=47; ns) | -7 (-24-22; n=23; ns) |
| **NGAL** | 29 (-20-136; n=35; **p=0.010)** | 13 (-88-106; n=47; ns) | 28 (-41-109; n=23; ns) |
| **Osteonectin** | -30 (-310 – 131; n=35; ns) | 10 (-270 – 210; n=47; ns) | -40 (-250 – 260; n=23; ns) |
| **Resistin** | 240 (-480 – 1050; n=35; ns) | 404 (-610 – 1270; n=47; ns) | -200 (-1040 – 970; n=23; ns) |
| **Leptin/Adiponection** | 0.017 (-0.014 – 0.110; n=35; **p=0.042**) | 0.004 (-0.126 – 0.0069; n=47; ns) | -0.019 (-0.113 – 0.334; n=23; ns) |
| **Resistin/Adiponectin** | -0.050 (-0.216 – 0.125; n=35; ns) | -0.051 (-0.357 – 0.063; n=47; **p=0.020**) | -0.234 (-0.393 – -0.117; n=23; **p=0.0067**) |

Abbreviations: ns, not significant; BAFF, B-cell activating factor; FGF‑21, fibroblast growth factor 21; NGAL, neutrophil gelatinase-associated lipocalin.

**Supplementary Table S10.** Adipokine levels in patients sub-divided according to age of onset, early (<12 years of age) vs late (≥12 years of age).

|  | **Early onset asthma**  (<12 years of age)  n=25 | **Late onset asthma**  (≥12 years of age)  n=85 | **P value**  Mann Whitney or Chi^2^ test. |
| --- | --- | --- | --- |
| Asthma severity | 61.8% MMA, 38.2% SA | 38.5% MMA, 61.5% SA | **0.017** |
| Sex | 47.1% male, 52.9% female | 40.4% male, 59.6% female | 0.490 |
| BMI | 24.65 (22.55–28.00) | 26.70 (24.00–29.80) | **0.0268** |
| Adiponectin | 5080.00 (4070.00–5680.00) | 5170.00 (4130.00–5860.00) | 0.9829 |
| Adipsin | 3310.00 (2720.00–3670.00) | 3680.00 (3190.00–4360.00) | **0.0027** |
| BAFF | 229.00 (208.00–265.00) | 235.00 (186.00–278.00) | 0.6302 |
| FGF-21 | 128.00 (115.00–138.00) | 144.00 (125.00–165.00) | **0.0035** |
| Leptin | 1960.00 (367.00–3990.00) | 1670.00 (679.00–3860.00) | 0.8978 |
| Lipocalin | 241.00 (193.50–325.00) | 236.00 (166.00–310.00) | 0.8557 |
| Osteonectin | 1620.00 (1300.00–1740.00) | 1680.00 (1370.00–1990.00) | 0.1642 |
| Resistin | 5700.00 (4820.00–8170.00) | 6430.00 (4780.00–7460.00) | 0.7107 |
| Chemerin | 111.00 (95.00–128.00) | 119.00 (100.00–161.00) | 0.3294 |

**Supplementary Table S11.** Change in adipokines, clinical and physiological parameters during the medication optimization phase Visit 1 to Visit 2.

|  | **Mild to moderate asthma**  **Change (range; N; p-value)** | **Severe asthma**  **Change (range; N; p-value)** |
| --- | --- | --- |
| **Leptin** | -2.0 (-285 - 249; n=50; ns) | 9.5 (-555 - 391; n=62; ns) |
| **Adiponectin** | 125 (-372 - 488; n=50; ns) | 10.0 (-410 - 630; n=62; ns) |
| **Adipsin** | 35 (-172 - 420; n=50; ns) | -180 (-642 - 360; n=62; **p=0.026)** |
| **BAFF** | 0.5 (-32.2 – 28.2; n=50; ns) | -9.0 (-41.0 – 24.5; n=62; ns) |
| **Chemerin** | 2.5 (-20.8 – 18.5; n=30; ns) | -5.0 (-22.5 – 12.0; n=37; ns) |
| **FGF-21** | 1.5 (-15.0 – 19.5; n=50; ns) | -6.5 (-24.2 – 11.2; n=62; **p=0.038**) |
| **NGAL** | -10.5 (-62.4 – 75.8; n=50; ns) | -3.0 (-62.2 – 76.3; n=62; ns) |
| **Osteonectin** | 3.5 (-142 – 172; n=50; ns) | 5.0 (-195 – 212; n=62; ns) |
| **Resistin** | 110 (-408 – 908; n=50; ns) | -116 (-1120 – 775; n=62; ns) |
| **FVC (% predicted)** | 0.18 (-4.3 – 3.5; n=51; ns) | 1.37 (-5.9 – 6. 9; n=70; ns) |
| **FEV1/FVC** | 0.030 (0.008 – 0.060; n=51; **p<0.0001**) | 0.010 (-0.036 – 0.048; n=70; ns) |
| **FEV1 (% predicted)** | 3.8 (-1.8 – 8.9; n=51; **p=0.0009**) | 0.8 (-5.0 – 7.5; n=70; ns) |
| **ACQ6** | -0.17 (-0.50 – 0.00; n=53; **p<0.0001**) | 0.00 (-0.34 – 0.33; n=64; ns) |
| **SGRQ** | -5.6 (-8.7 – 1.8; n=39; **p=0.0025**) | -1.0 (-7.8 – 5.5; n=60; ns) |
| **Blood eosinophils** | 0 (-90 – 70; n=49; ns) | 0 (-90 – 80; n=66; ns) |
| **Blood neutrophils** | 40 (-420 – 470; n=47; ns) | -100 (-1010 – 560; n=65; ns) |
| **Sputum Eosinophils (%)** | -0.2 (-1.8 – 0.7; n=34; ns) | 0 (-3.3 – 1.3; n=38; ns) |
| **Sputum Neutrophils (%)** | 0.2 (-10.3 – 9.4; n=34; ns) | 4.6 (-8.0 – 23.1; n=38; ns) |
| **FeNO** | -7.4 (-17.1 - -0.1; n=26; **p=0.0024**) | -2.2 (-30.6 – 5.4; n=28; ns) |

Abbreviations: ns, not significant; BAFF, B-cell activating factor; FGF‑21, fibroblast growth factor 21; NGAL, neutrophil gelatinase-associated lipocalin.

**Supplementary Figure S1.** Effect of two weeks of oral prednisolone on A) eosinophils, B) neutrophils, C) FVC and D) FEV_1_ in patients with severe or mild to moderate asthma, where results are shown as paired individual data points (Wilcoxon matched-pairs signed rank test, *p<0.05, **p<0.01, ***p<0.001 and ****p<0.0001).

A

B

C

D

**References**

1. The ENFUMOSA cross-sectional European multicentre study of the clinical phenotype of chronic severe asthma. European Respiratory Journal 2003; 22: 470.

2. Bel EH, Sousa A, Fleming L, Bush A, Chung KF, Versnel J, Wagener AH, Wagers SS, Sterk PJ, Compton CH. Diagnosis and definition of severe refractory asthma: an international consensus statement from the Innovative Medicine Initiative (IMI). Thorax 2011; 66: 910-917.

3. Bousquet J, Mantzouranis E, Cruz AA, Aït-Khaled N, Baena-Cagnani CE, Bleecker ER, Brightling CE, Burney P, Bush A, Busse WW, Casale TB, Chan-Yeung M, Chen R, Chowdhury B, Chung KF, Dahl R, Drazen JM, Fabbri LM, Holgate ST, Kauffmann F, Haahtela T, Khaltaev N, Kiley JP, Masjedi MR, Mohammad Y, O'Byrne P, Partridge MR, Rabe KF, Togias A, van Weel C, Wenzel S, Zhong N, Zuberbier T. Uniform definition of asthma severity, control, and exacerbations: document presented for the World Health Organization Consultation on Severe Asthma. J Allergy Clin Immunol 2010; 126: 926-938.

4. Moore WC, Bleecker ER, Curran-Everett D, Erzurum SC, Ameredes BT, Bacharier L, Calhoun WJ, Castro M, Chung KF, Clark MP, Dweik RA, Fitzpatrick AM, Gaston B, Hew M, Hussain I, Jarjour NN, Israel E, Levy BD, Murphy JR, Peters SP, Teague WG, Meyers DA, Busse WW, Wenzel SE, National Heart, Lung Blood Institute's Severe Asthma Research Program. Characterization of the severe asthma phenotype by the National Heart, Lung, and Blood Institute's Severe Asthma Research Program. The Journal of allergy and clinical immunology 2007; 119: 405-413.

5. Quanjer PH, Tammeling GJ, Cotes JE, Pedersen OF, Peslin R, Yernault JC. Lung volumes and forced ventilatory flow. Report working party standardisation lung function tests, European Community for Steel and Coal. Official statement of the European Respiratory Society. Eur Respir J 1993; 16:5-40.

6. Okamoto M, Hoshino T, Kitasato Y, Sakazaki Y, Kawayama T, Fujimoto K, Ohshima K, Shiraishi H, Uchida M, Ono J, Ohta S, Kato S, Izuhara K, Aizawa H. Periostin, a matrix protein, is a novel biomarker for idiopathic interstitial pneumonias. Eur Respir J 2011;37:1119-1127.

7. Paggiaro PL, Chanez P, Holz O, Ind PW, Djukanovic R, Maestrelli P, Sterk PJ. Sputum induction. Eur Repir J 2002 ; 20 Suppl 37 :3s-8s.

8. Recommendations for standardized procedures for the on-line and off-line measurement of exhaled lower respiratory nitric oxide and nasal nitric oxide in adults and children. Official statement by the ATS. Am J Respir Crit Care Med 1999; 160:2104-17.

9. Dweik RA, Boggs PB, Erzurum SC, Irvin CG et al. An official ATS clinical practice guideline: interpretation of exhaled nitric oxide levels (FENO) for clinical applications. Am J Respir Crit Care Med 2011; 184:602-15.

10. Okamoto M, Hoshino T, Kitasato Y, Sakazaki Y, Kawayama T, Fujimoto K, Ohshima K, Shiraishi H, Uchida M, Ono J, Ohta S, Kato S, Izuhara K, Aizawa H. Periostin, a matrix protein, is a novel biomarker for idiopathic interstitial pneumonias. Eur Respir J 2011;37:1119-1127.
